# Impact of Aging on the Frequency, Phenotype, and Function of CD161-Expressing T Cells

**DOI:** 10.3389/fimmu.2018.00752

**Published:** 2018-04-19

**Authors:** Kornelis S. M. van der Geest, Bart-Jan Kroesen, Gerda Horst, Wayel H. Abdulahad, Elisabeth Brouwer, Annemieke M. H. Boots

**Affiliations:** ^1^Department of Rheumatology and Clinical Immunology, University of Groningen, University Medical Center Groningen, Groningen, Netherlands; ^2^Department of Laboratory Medicine, University of Groningen, University Medical Center Groningen, Groningen, Netherlands; ^3^Department of Pathology and Medical Biology, University of Groningen, University Medical Center Groningen, Groningen, Netherlands

**Keywords:** aging, T lymphocytes subsets, CD161, cytokines, T helper 1 cells, T helper 17 cells, mucosal-associated invariant T cell

## Abstract

Immune-aging is associated with perturbed immune responses in the elderly. CD161-expressing T cells, i.e., the previously described subsets of CD161^+^ CD4^+^ T cells, CD161^high^ CD8^+^ T cells, and CD161^int^ CD8^+^ T cells, are highly functional, pro-inflammatory T cells. These CD161-expressing T cells are critical in immunity against microbes, while possibly contributing to autoimmune diseases. So far, little is known about the impact of aging on the frequency, phenotype, and function of these CD161-expressing T cells. In the current study, we investigated the impact of aging on CD161^+^ CD4^+^ T cells, CD161^high^ CD8^+^ T cells, and CD161^int^ CD8^+^ T cells in peripheral blood samples of 96 healthy subjects (age 20–84). Frequencies of CD161^+^ CD4^+^ T cells and CD161^int^ CD8^+^ T cells were stable with aging, whereas frequencies of CD161^high^ CD8^+^ T cells declined. Although CD161^high^ CD8^+^ T cells were mostly T cell receptor-Vα7.2^+^ mucosal-associated invariant T cells, CD161 expressing CD4^+^ and CD8^+^ T cells showed a limited expression of markers for gamma–delta T cells or invariant natural killer (NK) T cells, in both young and old subjects. In essence, CD161-expressing T cells showed a similar memory phenotype in young and old subjects. The expression of the inhibitory NK receptor KLRG1 was decreased on CD161^+^ CD4^+^ T cells of old subjects, whereas the expression of other NK receptors by CD161-expressing T cells was unaltered with age. The expression of cytotoxic effector molecules was similar in CD161^high^ and CD161^int^ CD8^+^ T cells of young and old subjects. The ability to produce pro-inflammatory cytokines was preserved in CD161^high^ and CD161^int^ CD8^+^ T cells of old subjects. However, the percentages of IFN-γ^+^ and interleukin-17^+^ cells were significantly lower in CD161^+^ CD4^+^ T cells of old individuals than those of young individuals. In addition, aging was associated with a decrease of nonclassic T helper 1 cells, as indicated by decreased percentages of CD161-expressing cells within the IFN-γ^+^ CD4^+^ T cell compartment of old subjects. Taken together, aging is associated with a numerical decline of circulating CD161^high^ CD8^+^ T cells, as well as a decreased production of pro-inflammatory cytokines by CD161^+^ CD4^+^ T cells. These aging-associated changes could contribute to perturbed immunity in the elderly.

## Introduction

Aging-associated perturbations of the immune system have been linked to increased risks for infections and autoimmune diseases in the elderly (1, 2). Insight into these immunological changes may eventually help to develop strategies to correct immunological disturbances in aged subjects.

The T cell compartment is especially affected by aging. The production of naive T cells shows a strong aging-associated decline, which starts already early in life (3). By contrast, a shift toward the memory T cell compartment develops upon antigenic stimulation by environmental stimuli (4). Consequently, the maintenance of the already existing naive and memory T cells is important to preserve immunity during adult life (5). Another aging-associated change in the T cell compartment of humans includes the acquisition of natural killer (NK) receptors and cytotoxic effector molecules by aged T cells (6, 7). In addition, the balance between pro-inflammatory and anti-inflammatory T cells is disturbed in the elderly (8). Thus, aging affects the numerical, phenotypic, and functional aspects of both the naive and memory T cell compartment in humans.

Cytomegalovirus (CMV) infection also has a broad impact on the T cell compartment (9). Latent infection with CMV is associated with skewing of the T cell receptor (TCR) repertoire (4). CMV drives the expansion of memory T cells, both in the circulation and in the peripheral tissues (10, 11). CMV-specific memory T cells may express NK receptors and are potent producers of pro-inflammatory cytokines, such as IFN-γ and tumor necrosis factor-α (TNF-α) (12–14). Furthermore, CMV has been linked to poor vaccination responses and a slightly increased mortality rate in the elderly (9, 15–17). Overall, CMV contributes to immune senescence in humans (9).

Ample evidence indicates that the expression of the C-type lectin receptor CD161 identifies subsets of CD4^+^ and CD8^+^ T cells with a strong pro-inflammatory phenotype (18–20). These CD161-expressing T cells are considered important for antimicrobial immunity in humans (20–24). However, CD161-expressing T cells may also contribute to the development of various autoimmune diseases (25–28). CD161 is expressed by CD4^+^ T helper 17 (Th17) lineage cells and nonclassic T helper 1 (Th1) cells (18, 29–31). In addition, two subsets of CD8^+^ T cells with different expression levels of CD161 have been identified (19, 20). Firstly, high expression levels of CD161 (CD161^high^) are found on a subset of innate-like CD8^+^ T cells termed mucosal-associated invariant T (MAIT) cells (32, 33). Another population of CD8^+^ T cells is characterized by intermediate expression levels of CD161 (CD161^int^) and contributes to antiviral immunity (20). Although several studies have shown that frequencies of MAIT cells decrease with age (34–36), little is known about the effect of aging on the other CD161-expressing T cell subsets.

In the current study, we assessed the impact of aging on the frequencies, phenotype, and function of CD161-expressing CD4^+^ and CD8^+^ T cells in the peripheral blood of humans. While taking into account CMV serostatus, we investigated the effect of age on the numbers of circulating CD161-expressing T cells in a large group of healthy subjects. Furthermore, we studied the expression of T cell differentiation markers, NK receptors, cytotoxic effector molecules, and pro-inflammatory cytokines by CD161-expressing T cells of young and old subjects. Taken together, our study provides a unique and comprehensive insight into the effect of aging on highly functional, pro-inflammatory T cell populations in humans.

## Materials and Methods

### Study Subjects and Samples

Blood samples were obtained from 96 healthy individuals (age range 20–84, of which 26 were males) Subjects underwent a thorough examination of their health status, as described previously (8). Exclusion criteria included infection, malignancy, autoimmune disease, chronic liver or kidney disease, alcohol or drug abuse, diabetes mellitus, current pregnancy, or immunosuppressive treatment. Written informed consent was obtained from all study participants. The study was approved by the Medical Ethical Committee of the UMCG. All procedures were in accordance with the Declaration of Helsinki.

### Flow Cytometry

Blood samples (EDTA) were stained with the following monoclonal antibodies: CD3-eFluor605, CD4-eFluor450, TCRγδ-PE (eBioscience), CD45RO-FITC, CCR7-PE-Cy7, CD4-PerCP, CD8-PerCP, CD8-APC-H7, DNAX accessory molecule-1 (DNAM-1)-FITC (BD), CD161-PE, CD161-APC (Miltenyi), 2B4-PE, NKG2D-PE-Cy7, TCR-Vα7.2-FITC, TCR-Vα24-Jα18-FITC (Biolegend), TCR-Vβ11-PE (Beckman Coulter), and KLRG1-FITC (generous gift from H. Pirchner). An overview of antibody panels is shown in Table S1 in Supplementary Material. Samples were subsequently treated with BD Lysing Solution (BD Biosciences) according to instructions of the manufacturer. Samples were measured on a LSR-II flow cytometer (BD) and analyzed with Kaluza Analysis Software (Beckman Coulter). In addition, the absolute numbers of circulating lymphocyte subsets were determined according to the MultiTest TruCount method (BD), as described by the manufacturer. Data were acquired on a FACSCanto-II flow cytometer (BD) and analyzed with FACSCanto Clinical Software (BD). The number of events for a particular T cell population needed to be more than 100 to allow for subsequent analysis of cellular markers, cytokines, and cytotoxic molecules.

### Intracellular Cytokine Staining

Blood samples (heparin) were diluted 1:1 with RPMI and stimulated with 40 nM PMA and 2 nM Ca^2+^ ionophore A23187 in the presence of 3 µM brefeldin A (all Sigma) for 4 h. Subsequently, red blood cells were lysed with ammonium chloride. The remaining cells were treated with Fix/Perm reagents A and B (Invitrogen) and stained with the following antibodies: CD3-eFluor 605, CD4-eFluor450, interleukin (IL)-17-AF488, Perforin-FITC (eBioscience), CD161-APC (Miltenyi), CD8-APC-H7, Granzyme-B-PE (BD), IFN-γ-PerCP-Cy5.5, IL-4-PE, and TNF-α-PerCP-Cy5.5 (Biolegend). Samples were measured on an LSR-II flow cytometer (BD) and analyzed with Kaluza Analysis Software (Beckman Coulter).

### Measurement of CMV-Specific IgG

As previously described (4), 96-well ELISA plates (Greiner) were coated overnight with lysates of CMV-infected fibroblasts. Lysates of non-infected fibroblasts were used as negative controls. Following the coating, diluted serum samples were incubated for 1 h. Goat anti-human IgG was added and incubated for 1 h. Samples were incubated with phosphatase for 15 min, and the reaction was stopped with NaOH. The plates were scanned on a Versamax reader (Molecular Devices). A pool of sera from three CMV-seropositive individuals with known concentrations of CMV-specific IgG was used to quantify CMV-specific IgG in the tested samples. Detailed information on CMV serostatus of the healthy donors is shown for experiments reporting the phenotype and function of CD161-expressing T cells in Table S2 in Supplementary Material.

### Statistics

The Mann–Whitney *U*-test was used to compare continuous variables between different age groups. Correlations were determined with Spearman’s rank correlation coefficient. Univariate and multivariate linear regression analyses were used to further determine the impact of age and CMV serostatus on CD161-expressing T cell counts in healthy subjects. Firstly, each variable was tested in a univariate linear regression analysis. Subsequently, variables with *p* < 0.3 in the univariate analysis were used in the multivariate analysis. Reported *B*-coefficients indicate how much cell counts (10^9^/L) change with every unit increase of the tested variable. The categorical variable CMV serostatus was assigned a value of 0 or 1. CMV: 0 = seronegative, 1 = seropositive. Non-normally distributed outcome variables were transformed (i.e., log or square root). Analysis was performed with SPSS 23.0 Software and GraphPad Prism 5.0. *P*-values less than 0.05 were considered to be significant.

## Results

### Characterization of Healthy Subjects

To investigate the impact of aging on CD161-expressing T cells, we recruited a group of healthy subjects with a wide age range. Detailed characteristics of the healthy subjects are shown in Table 1. Young (age 18–39), intermediate (age 40–59), and old (age ≥60) subjects showed a similar distribution of gender and CMV serostatus. Furthermore, general laboratory parameters were comparable among the different age groups.

**Table 1:** Subject characteristics.

	Young (age 18–39)	Intermediate (age 40–59)	Old (age ≥60)
No.	22	24	50
Age, years, median (range)	27 (20–38)	57 (40–59)	69 (60–84)
No. male (%)	6 (27)	6 (25)	14 (28)
No. CMV positive (%)	12 (55)	11 (46)	27 (54)
Hemoglobin, mmol/L, median (range)^a^	8.3 (7.3–9.5)	8.5 (7.5–10.0)	8.6 (7.1–10.2)
Leukocyte count, 10^9^/L, median (range)^a^	4.7 (2.8–7.5)	5.3 (3.5–8.2)	5.8 (2.9–9.1)
Thrombocyte count, 10^9^/L, median (range)^a^	275 (173–422)	240 (161–381)	240 (121–350)
ESR, mm/h, median (range)^a^	6 (1–16)	8 (1–15)	8 (2–26)
Creatinine, μmol/L, median (range)^a^	62 (53–90)	71 (58–89)	69 (49–97)

*No., number; ESR, erythrocyte sedimentation rate*.

*^a^Performed in 11/22 young individuals, 19/24 intermediate age individuals, and 50/50 aged individuals*.

### Aging Is Associated With a Decrease of Circulating CD161^high^ CD8^+^ T Cells

**Figure 1:**
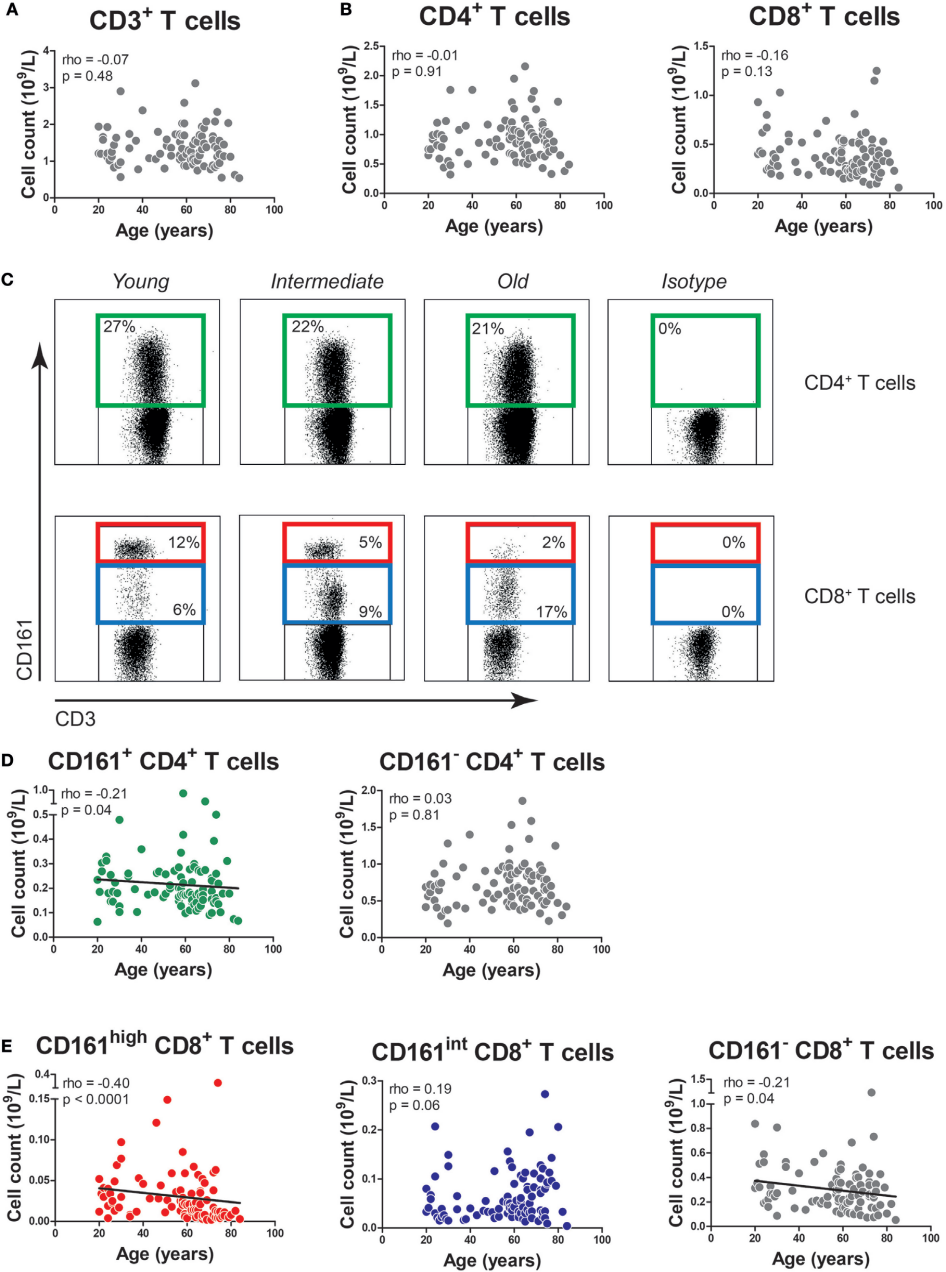
The effect of age on the absolute numbers of CD161-expressing T cells. **(A)** The absolute numbers of CD3^+^ T cells, as well as **(B)** CD4^+^ T cells and CD8^+^ T cells in the peripheral blood of 96 healthy subjects (age range 20–84). **(C)** Flow cytometric gating of CD161^+^ CD4^+^ T cells, CD161^high^ CD8^+^ T cells, and CD161^int^ CD8^+^ T cells in the peripheral blood of healthy young, intermediate, and old subjects. **(D)** The absolute numbers of CD161^+^ CD4^+^ T cells, CD161^−^ CD4^+^ T cells, and **(E)** CD161^high^ CD8^+^ T cells, CD161^int^ CD8^+^ T cells, and CD161^−^ CD8^+^ T cells in the peripheral blood of same donors as mentioned at **(A,B)**. Statistical significance by Spearman’s rank test is shown.

Firstly, we determined the impact of age on total CD3^+^, CD4^+^, and CD8^+^ T cell numbers in our study population. The numbers of CD3^+^ T cells, CD4^+^ T cells, and CD8^+^ T cells were stable with age (Figures 1A,B).

Next, we studied the effect of age on the numbers of CD161-expressing T cells. In accordance with prior studies (18–20), we identified CD161^+^ cells within the CD4^+^ T cell compartment, as well as CD8^+^ T cells with high and intermediate expression levels of CD161, i.e., CD161^high^ and CD161^int^ cells (Figure 1C). The absolute numbers of CD161^+^ CD4^+^ T cells showed a slight decrease with age (*p* = 0.04), whereas the absolute numbers of CD161^−^ CD4^+^ T cells remained stable (Figure 1D). The percentages of CD161^+^ CD4^+^ T cells showed a statistically significant inverse correlation with age (Figure S1A in Supplementary Material). The absolute numbers of CD161^high^ and CD161^−^ CD8^+^ T cells showed a negative correlation with age, while the absolute numbers of CD161^int^ CD8^+^ T cells remained stable (Figure 1E). Percentages of CD161^high^ CD8^+^ T cells were also negatively associated with age (Figure S1B in Supplementary Material). However, due to the absolute decrease of CD161^high^ and CD161^−^ CD8^+^ T cells, the percentage of CD161^int^ CD8^+^ T cells increased with age. These findings suggest that aging might impact the numbers of CD161-expressing T cells.

Subsequently, we performed a multivariate analysis in order to evaluate the impact of both aging and CMV serostatus on the absolute numbers of CD161-expressing T cells. In this analysis, neither aging nor CMV showed an effect on the numbers of CD161^+^ and CD161^−^ CD4^+^ T cells (Table 2). Aging was associated with declining numbers of CD161^high^ CD8^+^ T cells, whereas CMV seropositivity was linked to a higher number of CD161^int^ CD8^+^ T cells. Independent effects of aging and CMV serostatus were also observed on the absolute numbers of CD161^−^ CD8^+^ T cells. Taken together, aging is associated with a decrease of CD161^high^ CD8^+^ T cells, whereas the absolute numbers of CD161^int^ CD8^+^ T cells and CD161^+^ CD4^+^ T cells remain stable.

**Table 2:** Univariate and multivariate linear regression analysis for the absolute numbers of CD161-expressing T cell subsets.

Dependent variable	Predicting variables	Univariate analysis B (95% CI)	*p*-value	Multivariate analysis B (95% CI)	*p*-value
CD161^+^ CD4^+^ (10^9^/L)	Age	−0.001 (−0.002 to 0.000)	0.176	−0.001 (−0.002 to 0.000)^a^	0.158
	CMV	0.027 (−0.008 to 0.068)	0.133	0.034 (−0.008 to 0.084)^a^	0.120

CD161^−^ CD4^+^ (10^9^/L)	Age	0.001 (−0.002 to 0.004)	0.426	^†^	
	CMV	0.030 (−0.083 to 0.152)	0.614	^†^	

CD161^high^ CD8^+^ (10^9^/L)	Age	−0.001 (−0.002 to 0.000)	0.001	−0.001 (−0.002 to 0.000)^b^	0.001
	CMV	−0.004 (−0.010 to 0.004)	0.294	−0.010 (−0.026 to 0.012)^b^	0.318

CD161^int^ CD8^+^ (10^9^/L)	Age	0.000 (0.000 to 0.001)	0.156	0.000 (0.000 to 0.000)^c^	0.159
	CMV	0.030 (0.014 to 0.051)	0.000	0.021 (0.010 to 0.037)^c^	0.000

CD161^−^ CD8^+^ (10^9^/L)	Age	−0.003 (−0.006 to −0.001)	0.016	−0.003 (−0.005 to −0.001)^d^	0.006
	CMV	0.122 (0.059 to 0.200)	0.000	0.199 (0.101 to 0.320)^d^	0.000

*Data are obtained for 96 healthy individuals (age range 20–84). Firstly, each variable was tested in a univariate linear regression analysis. Subsequently, variables with *p* < 0.3 in the univariate analysis were used in the multivariate analysis. Reported *B*-coefficients indicate how much cell counts (10^9^/L) change with every unit increase of the predicting variable. The categorical variable CMV was assigned a value of 0 or 1, where 0 = CMV seronegative and 1 = CMV seropositive. Age: in years. CI = confidence interval*.

*^a^Model R^2^ = 0.045*.

*^b^Model R^2^ = 0.120*.

*^c^Model R^2^ = 0.184*.

*^d^Model R^2^ = 0.233*.

*^†^Not tested in multivariate analysis due to p > 0.3 in univariate regression analysis*.

### Aging Does Not Affect the Expression of Innate-Like T Cell Markers by CD161-Expressing T Cells

**Figure 2:**
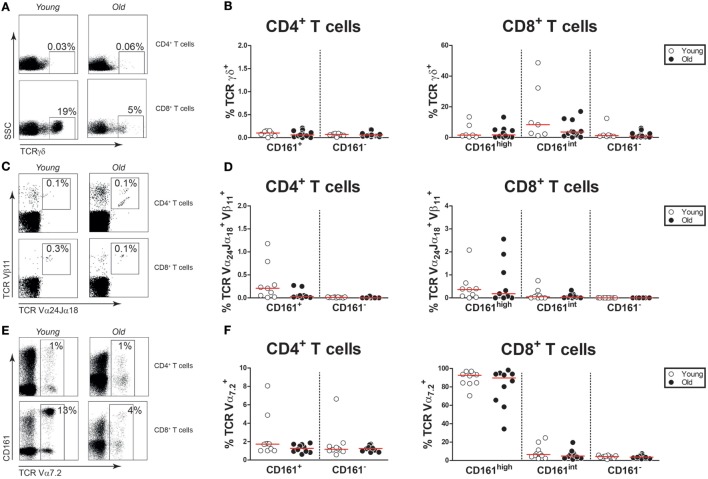
Innate-like T cell markers on CD161-expressing T cells. **(A)** Flow cytometric gating of TCRγδ^+^ cells in the CD4^+^ and CD8^+^ T cell compartment of a young and old subject. **(B)** Percentages of TCRγδ^+^ cells within the CD161-defined CD4^+^ and CD8^+^ T cell subsets of 7 young [of which 4 were cytomegalovirus (CMV) seropositive] and 16 old (of which 8 were CMV seropositive) subjects. **(C)** Flow cytometric gating of TCR-Vα24-Jα18^+^TCR-Vβ11^+^ cells in the CD4^+^ and CD8^+^ T cell compartment of a young and old subject. **(D)** Percentages of TCR-Vα24-Jα18^+^ TCR-Vβ11^+^ cells within the CD161-defined CD4^+^ and CD8^+^ T cell subsets of nine young (of which four were CMV seropositive) and nine old (of which four were CMV seropositive) subjects. **(E)** Flow cytometric gating of TCR-Vα7.2^+^ cells in the CD4^+^ and CD8^+^ T cell compartment of a young and old subject. **(F)** Percentages of TCR-Vα7.2^+^ cells within the CD161-defined CD4^+^ and CD8^+^ T cell subsets of 10 young (of which 5 were CMV seropositive) and 10 old (of which 5 were CMV seropositive) subjects. Graphs in which CMV-seropositive young and old subjects are highlighted in the figures are shown in Figure S2 in Supplementary Material.

Previous studies have reported that gamma–delta T cells, invariant natural killer T (iNKT) cells, and MAIT cells may express CD161 (33, 37, 38). Therefore, we investigated if CD161-expressing T cells of young and old subjects express markers defining these innate-like T cells. Gamma–delta T cells were identified by the expression of the TCRγδ receptor (Figure 2A) in young and old subjects with comparable CMV-seropositivity rates (Figure S2 in Supplementary Material). The TCRγδ receptor was present on few CD161^+^ CD4^+^ T cells, both in young and in old subjects (Figure 2B). Percentages of TCRγδ^+^ cells were limited within the CD161^high^ and CD161^int^ CD8^+^ T cell subsets, and no differences were observed between young and old subjects. iNKT cells were identified as TCR-Vα24-Jα18 and TCR-Vβ11 co-expressing cells (Figure 2C). Overall, percentages of iNKT cells were low and similar among the CD161-expressing T cell subsets of young and old subjects (Figure 2D). To delineate MAIT cells, additional staining was performed for the TCR-Vα7.2 receptor (Figure 2E). Although limited expression of TCR-Vα7.2 was observed among all CD161-defined T cell populations, the expression of this receptor was largely restricted to CD161^high^ CD8^+^ T cells, with similar percentages observed in young and old subjects (Figure 2F). Overall, the expression of innate-like T cell markers by CD161-expressing CD4^+^ and CD8^+^ T cells was not affected by age.

**Figure 3:**
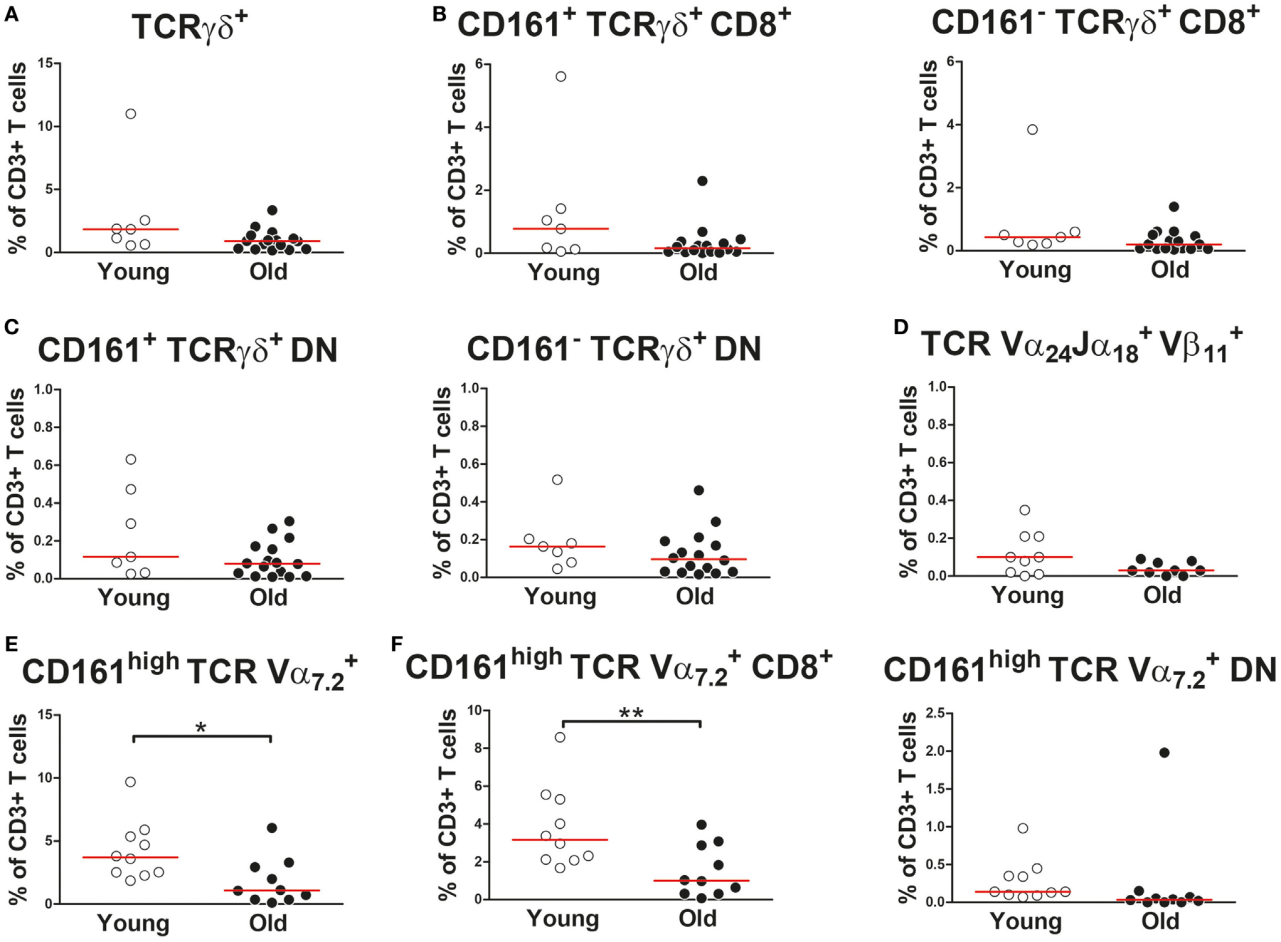
Proportions of CD161-expressing innate-like T cells in the CD3^+^ T cell compartment **(A)** Percentages of total TCRγδ^+^ cells (i.e., gamma–delta T cells) [**(B)** left panel], CD161^+^ CD8^+^ gamma–delta T cells [**(B)** right panel], CD161^−^ CD8^+^ gamma–delta T cells [**(C)**, left panel], CD161^+^ double-negative (DN) gamma–delta T cells, and [**(C)**, right panel] CD161^−^ DN gamma–delta T cells within the CD3^+^ T cell compartment of 7 young [of which 4 were cytomegalovirus (CMV) seropositive] and 16 old (of which 8 were CMV seropositive) subjects. **(D)** Percentages of TCR-Vα24-Jα18^+^TCR-Vβ11^+^ cells (i.e., invariant natural killer T cells) within the CD3^+^ T cell compartment of nine young (of which four were CMV seropositive) and nine old (of which four were CMV seropositive) subjects. **(E)** Percentages of total CD161^high^ TCR-Vα7.2^+^ mucosal-associated invariant T (MAIT) cells, [**(F)**, left panel] CD8^+^ MAIT cells, and [**(F)**, right panel] DN MAIT cells within the CD3^+^ T cell compartment of 10 young (of which 5 were CMV seropositive) and 10 old (of which 5 were CMV seropositive) subjects. Statistical significance by Mann–Whitney *U*-test is shown as *p < 0.05 or **p < 0.01. Graphs in which CMV-seropositive young and old subjects are highlighted in the figures are shown in Figure S3 in Supplementary Material.

We subsequently investigated the effect of aging on proportions of the CD161-expressing innate-like T cell subsets within the total CD3^+^ T cell compartment. Percentages of TCRγδ^+^ cells, i.e., gamma–delta T cells, were similar in young and old subjects (Figure 3A). The same was true for percentages of CD161^+^ TCRγδ^+^ CD8^+^ T cells (Figure 3B). CD161^+^ TCRγδ^+^ CD4^+^ T cells were nearly absent, both in young and in old subjects (data not shown). However, a small portion of TCRγδ^+^ cells showed a CD4/CD8 double-negative (DN) phenotype. No difference was observed for percentages of CD161^+^ TCRγδ^+^ DN T cells within the CD3^+^ T cell pool of young and old subjects (Figure 3C). Proportions of TCR-Vα24-Jα18 and TCR-Vβ11 co-expressing cells, i.e., iNKT cells, were comparable in young and old subjects (Figure 3D). The low frequencies of iNKT cells precluded any further sub-analyses of these cells. Percentages of CD161^high^ TCR-Vα7.2^+^ MAIT cells were lower in the CD3^+^ T cell compartment of old subjects than that of young subjects (Figure 3E). This decrease resulted from a decrease of CD8^+^ MAIT cells rather than DN MAIT cells (Figure 3F). As previously reported by others (32, 35), CD4^+^ MAIT cells were nearly absent (data not shown). Thus, aging is associated with a decrease of CD8^+^ MAIT cells.

### Aging Is Not Associated With Major Changes in the Memory Phenotype of CD161-Expressing T Cells

**Figure 4:**
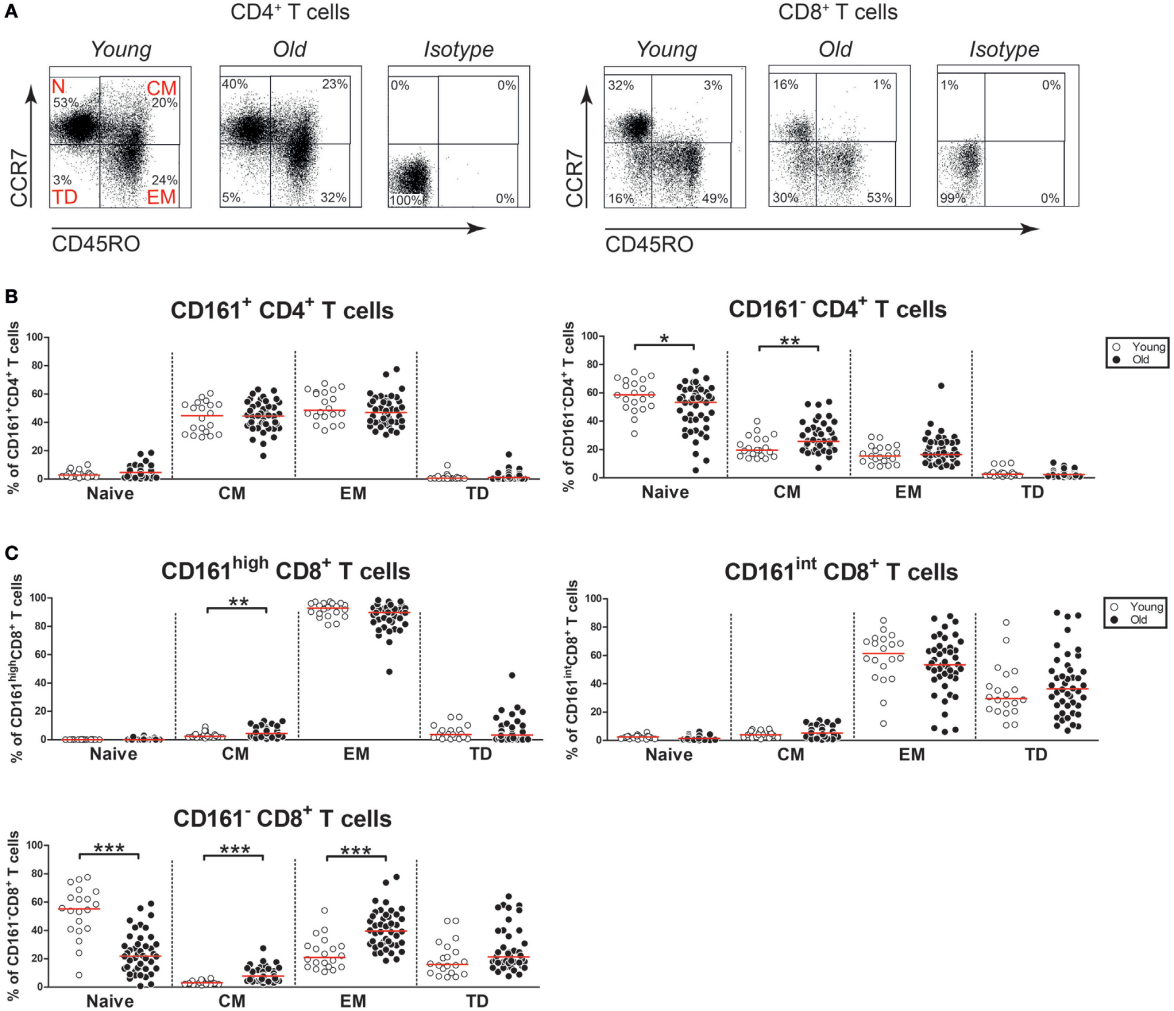
Differentiation markers on CD161-expressing T cells. **(A)** Flow cytometric gating of naive cells (N; CD45RO^−^CCR7^+^), central memory cells (CM) (CD45RO^+^CCR7^+^), effector memory cells (EM) (CD45RO^+^CCR7^−^), and terminally differentiated cells (TD) (CD45RO^−^CCR7^−^) within the CD4^+^ and CD8^+^ T cell compartment of a young and old subject. **(B)** Percentages of differentiation subsets within the CD161-defined CD4^+^ T cell subsets of 20 young [of which 10 were cytomegalovirus (CMV) seropositive] and 44 old (of which 22 were CMV seropositive) subjects. **(C)** Percentages of differentiation subsets within the CD161-defined CD8^+^ T cell subsets of the same donors as mentioned at **(B)**. Statistical significance by Mann–Whitney *U*-test is shown as *p < 0.05, **p < 0.01, or ***p < 0.001. Graphs in which CMV-seropositive young and old subjects are highlighted in the figures are shown in Figure S4 in Supplementary Material.

Next, we questioned if aging would impact the differentiation status of CD161-expressing CD4^+^ and CD8^+^ T cells. Based on the expression of CD45RO and CCR7, we identified naive (N; CD45RO^−^CCR7^+^), central memory (CM) (CD45RO^+^CCR7^+^), effector memory (EM) (CD45RO^+^CCR7^−^), and terminally differentiated (TD) (CD45RO^−^CCR7^−^) cells in the peripheral blood of the healthy subjects (Figure 4A). CD161^+^ CD4^+^ T cells were predominantly CM and EM cells, irrespective of age (Figure 4B). Nearly all CD161^high^ CD8^+^ T cells showed an EM cell phenotype (Figure 4C). Similar percentages of EM cells were observed among CD161^high^ CD8^+^ T cells of young and old subjects, whereas a small increase of CM cells was observed in the old. CD161^int^ CD8^+^ T cells were mostly EM and TD cells, with similar percentages in young and old subjects. In contrast to the relatively stable differentiation phenotype of CD161-expressing T cells, a clear shift from naive toward memory cells was observed among the CD161^−^ CD4^+^ and CD161^−^ CD8^+^ T cell fractions (Figures 4B,C). Thus, aging showed no substantial effect on the differentiation status of CD161-expressing T cells.

### Aging Has Limited Effect on NK Receptor Expression by CD161-Expressing T Cells

**Figure 5:**
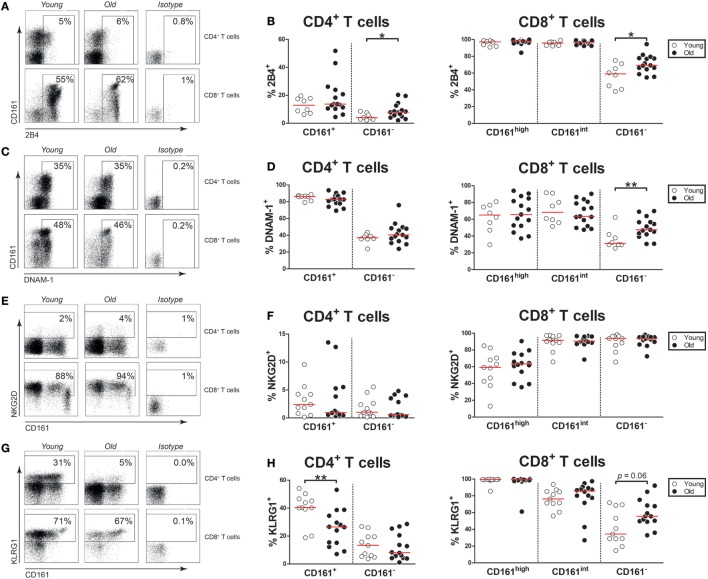
Natural killer markers on CD161-expressing T cells. **(A)** Flow cytometric gating of 2B4^+^ cells within the CD4^+^ and CD8^+^ T cell compartment of a young and old subject. **(B)** Percentages of 2B4^+^ cells within the CD161-defined CD4^+^ and CD8^+^ T cell subsets of 8 young [of which 4 were cytomegalovirus (CMV) seropositive] and 15 old (of which 7 were CMV seropositive) subjects. **(C)** Flow cytometric gating of DNAX accessory molecule-1 (DNAM-1)^+^ cells within the CD4^+^ and CD8^+^ T cell compartment of a young and old subject. **(D)** Percentages of DNAM-1^+^ cells within the CD161-defined CD4^+^ and CD8^+^ T cell subsets of the same subjects as shown in **(B)**. **(E)** Flow cytometric gating of NKG2D^+^ cells within the CD4^+^ and CD8^+^ T cell compartment of a young and old subject. **(F)** Percentages of NKG2D^+^ cells within the CD161-defined CD4^+^ and CD8^+^ T cell subsets of 11 young (of which 6 were CMV seropositive) and 14 old (of which 8 were CMV seropositive) subjects. **(G)** Flow cytometric gating of KLRG1^+^ cells within the CD4^+^ and CD8^+^ T cell compartment of a young and old subject. **(H)** Percentages of KLRG1^+^ cells within the CD161-defined CD4^+^ and CD8^+^ T cell subsets of the same subjects as shown in **(F)**. Statistical significance by Mann–Whitney *U*-test is shown as *p < 0.05 or **p < 0.01. Graphs in which CMV-seropositive young and old subjects are highlighted in the figures are shown in Figure S5 in Supplementary Material.

As aging is associated with the expression of NK receptors by T cells (6, 7), we next investigated the expression of NK receptors on CD161-expressing CD4^+^ and CD8^+^ T cells of young and old subjects. Firstly, we evaluated the activating NK receptor 2B4 on the CD4^+^ and CD8^+^ T cell subsets (Figure 5A). Few CD161^+^ CD4^+^ T cells expressed 2B4, without any difference between young and old subjects (Figure 5B). Nearly all CD161^high^ and CD161^int^ CD8^+^ T cells showed the expression of 2B4, irrespective of age. Next, we studied the activating NK receptor DNAM-1 (Figure 5C). The majority of CD161^+^ CD4^+^ T cells, CD161^high^ CD8^+^ T cells, and CD161^int^ CD8^+^ T cells expressed DNAM-1 (Figure 5D). Among these T cell subsets, the percentages of DNAM-1^+^ cells were similar in young and old subjects. Subsequently, the expression of the activating receptor NKG2D was evaluated (Figure 5E). Equally low percentages of NKG2D^+^ cells were observed among CD161^+^ CD4^+^ T cells of young and old individuals (Figure 5F). A significant proportion of CD161^high^ CD8^+^ T cells and nearly all CD161^int^ CD8^+^ T cells expressed NKG2D, with similar percentages in young and old subjects. Next, we evaluated the expression of the inhibitory receptor KLRG1 (Figure 5G). A significant portion of CD161^+^ CD4^+^ T cells expressed KLRG1 in young subjects, but the percentage of KLRG1^+^ cells was significantly lower in CD161^+^ CD4^+^ T cells of old subjects (Figure 5H). Most CD161^high^ and CD161^int^ CD8^+^ T cells expressed KLRG1, with similar percentages in young and old subjects. Taken together, aging was associated with a decreased expression of KLRG1 on CD161^+^ CD4^+^ T cells, while the expression of the other NK receptors was stable.

### Aging Has Limited Effect on Cytotoxic Effector Molecule Expression by CD161-Expressing T Cells

**Figure 6:**
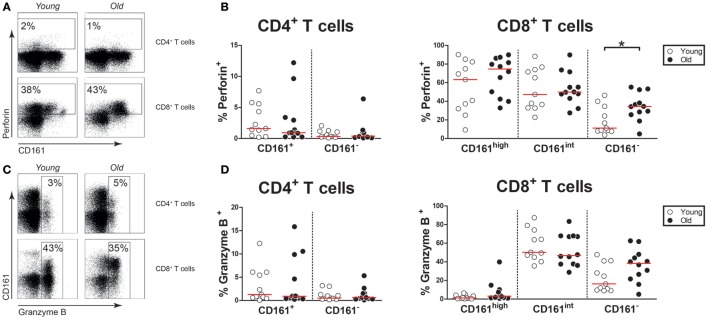
Cytotoxic effector molecules in CD161-expressing T cells. Intracellular staining for perforin and granzyme B was performed on non-stimulated blood samples. **(A)** Flow cytometric gating of perforin^+^ cells within the CD4^+^ and CD8^+^ T cell compartment of a young and old subject. **(B)** Percentages of perforin^+^ cells within the CD161-defined CD4^+^ and CD8^+^ T cell subsets of 11 young [of which 7 were cytomegalovirus (CMV) seropositive] and 12 old (of which 8 were CMV seropositive) subjects. **(C)** Flow cytometric gating of granzyme B^+^ cells within the CD4^+^ and CD8^+^ T cell compartment of a young and old subject. **(D)** Percentages of granzyme B^+^ cells within the CD161-defined CD4^+^ and CD8^+^ T cell subsets of the same subjects as shown in **(B)**. Statistical significance by Mann–Whitney *U*-test is shown as *p < 0.05. Graphs in which CMV-seropositive young and old subjects are highlighted in the figures are shown in Figure S6 in Supplementary Material.

Next, we questioned if aging would affect the expression of cytotoxic effector molecules, i.e., perforin and granzyme B, by CD161-expressing CD4^+^ and CD8^+^ T cells. Few CD161^+^ CD4^+^ T cells and approximately half of CD161^high^ and CD161^int^ CD8^+^ T cells expressed perforin (Figures 6A,B). In this respect, no difference was observed between young and old subjects. The expression of granzyme B was limited in CD161^+^ CD4^+^ T cells and CD161^high^ CD8^+^ T cells, whereas a significant portion of CD161^int^ CD8^+^ T cells expressed this cytotoxic effector molecule (Figures 6C,D). The expression of granzyme B was similar among the CD161-expressing subsets of young and old individuals. Overall, the expression of perforin and granzyme B among CD161-expressing cells was rather stable with age.

### Aging Is Associated With Decreased IFN-γ and IL-17 Expression by CD161^+^ CD4^+^ T Cells

**Figure 7:**
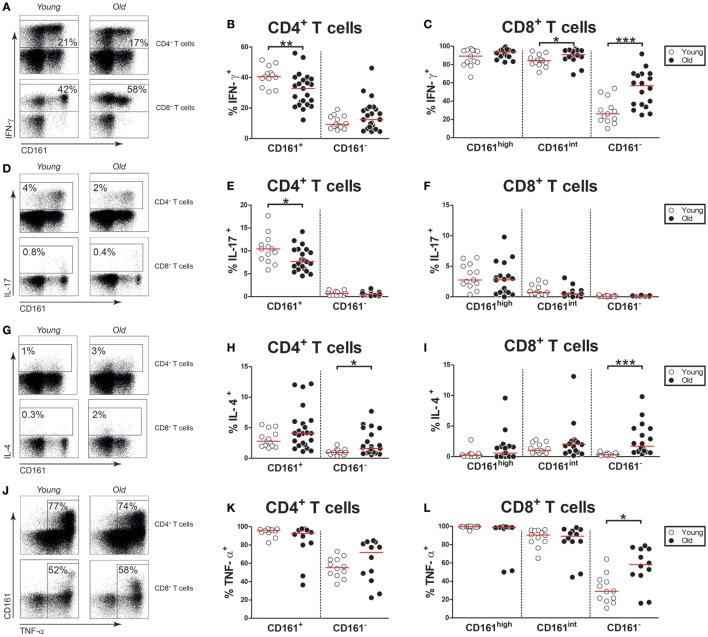
Cytokine expression by CD161-expressing T cells. Intracellular staining for pro-inflammatory cytokines was determined on blood samples that were stimulated with PMA and calcium ionophore in the presence of brefeldin A. **(A)** Flow cytometric gating of IFN-γ^+^ cells within the CD4^+^ and CD8^+^ T cell compartment of a young and old subject. **(B)** Percentages of IFN-γ^+^ cells within the CD161-defined CD4^+^ T cell subsets of 13 young [of which 8 were cytomegalovirus (CMV) seropositive] and 23 old (of which 15 were CMV seropositive) subjects. **(C)** Percentages of IFN-γ^+^ cells within the CD161-defined CD8^+^ T cell subsets of 13 young (of which 8 were CMV seropositive) and 18 old (of which 12 were CMV seropositive) subjects. **(D)** Flow cytometric gating of interleukin (IL)-17^+^ cells within the CD4^+^ and CD8^+^ T cell compartment of a young and old subject. **(E)** Percentages of IL-17^+^ cells within the CD161-defined CD4^+^ T cell subsets of the same subjects as shown in **(B)**. **(F)** Percentages of IL-17^+^ cells within the CD161-defined CD8^+^ T cell subsets of the same subjects as shown in **(C)**. **(G)** Flow cytometric gating of IL-4^+^ cells within the CD4^+^ and CD8^+^ T cell compartment of a young and old subject. **(H)** Percentages of IL-4^+^ cells within the CD161-defined CD4^+^ T cell subsets of the same subjects as shown in **(B)**. **(I)** Percentages of IL-4^+^ cells within the CD161-defined CD8^+^ T cell subsets of the same subjects as shown in **(C)**. **(J)** Flow cytometric gating of TNF-α^+^ cells within the CD4^+^ and CD8^+^ T cell compartment of a young and old subject. **(K)** Percentages of TNF-α^+^ cells within the CD161-defined CD4^+^ T cell subsets and **(L)** CD161-defined CD8^+^ T cell subsets of 12 young (of which 7 were CMV seropositive) and 12 old (of which 7 were CMV seropositive) subjects. Statistical significance by Mann–Whitney *U*-test is shown as *p < 0.05, **p < 0.01, or ***p < 0.001. Graphs in which CMV-seropositive young and old subjects are highlighted in the figures are shown in Figure S7 in Supplementary Material.

As CD161-expressing T cells are potent producers of pro-inflammatory cytokines, we investigated if aging impacts this function of CD161-expressing T cells. In contrast to CD161^−^ CD4^+^ and CD8^+^ T cells, a substantial portion of CD161^+^ CD4^+^ T cells and the vast majority of CD161^high^ and CD161^int^ CD8^+^ T cells were capable of producing IFN-γ upon PMA/Ca^2+^-ionophore stimulation (Figures 7A–C). Interestingly, percentages of IFN-γ producing cells were lower among CD161^+^ CD4^+^ T cells of old subjects than those of young subjects. By contrast, slightly more CD161^int^ CD8^+^ T cells were capable of producing IFN-γ in old subjects than young subjects. IL-17 was mostly produced by CD161^+^ CD4^+^ T cells and CD161^high^ CD8^+^ T cells (Figures 7D–F). Whereas the percentage of IL-17-producing cells was similar in CD161^high^ CD8^+^ T cells of young and old subjects, the percentage of IL-17^+^ cells was decreased in CD161^+^ CD4^+^ T cells of old individuals. A small fraction of CD161^+^ CD4^+^ T cells was capable of producing IL-4, with no differences between young and old subjects (Figures 7G,H). The expression of IL-4 was nearly absent in CD161^high^ and CD161^int^ CD8^+^ cells, irrespective of age (Figures 7G,I). Finally, we investigated CD161-expressing T cells for their ability to produce TNF-α (Figure 7J). Nearly all CD161^+^ CD4^+^ T cells, CD161^high^ CD8^+^ T cells, and CD161^int^ CD8^+^ T cells were capable of producing TNF-α (Figures 7K,L). Similar percentages of TNF-α^+^ cells were observed in these CD161-expressing T cell subsets of young and old subjects. Taken together, our findings confirm that CD161-expressing cells are potent producers of pro-inflammatory cytokines. However, CD161^+^ CD4^+^ T cells of old subjects show a diminished capacity to produce IFN-γ and IL-17 upon stimulation.

### Aging Is Associated With a Numerical Decline of Nonclassic Th1 Cells

**Figure 8:**
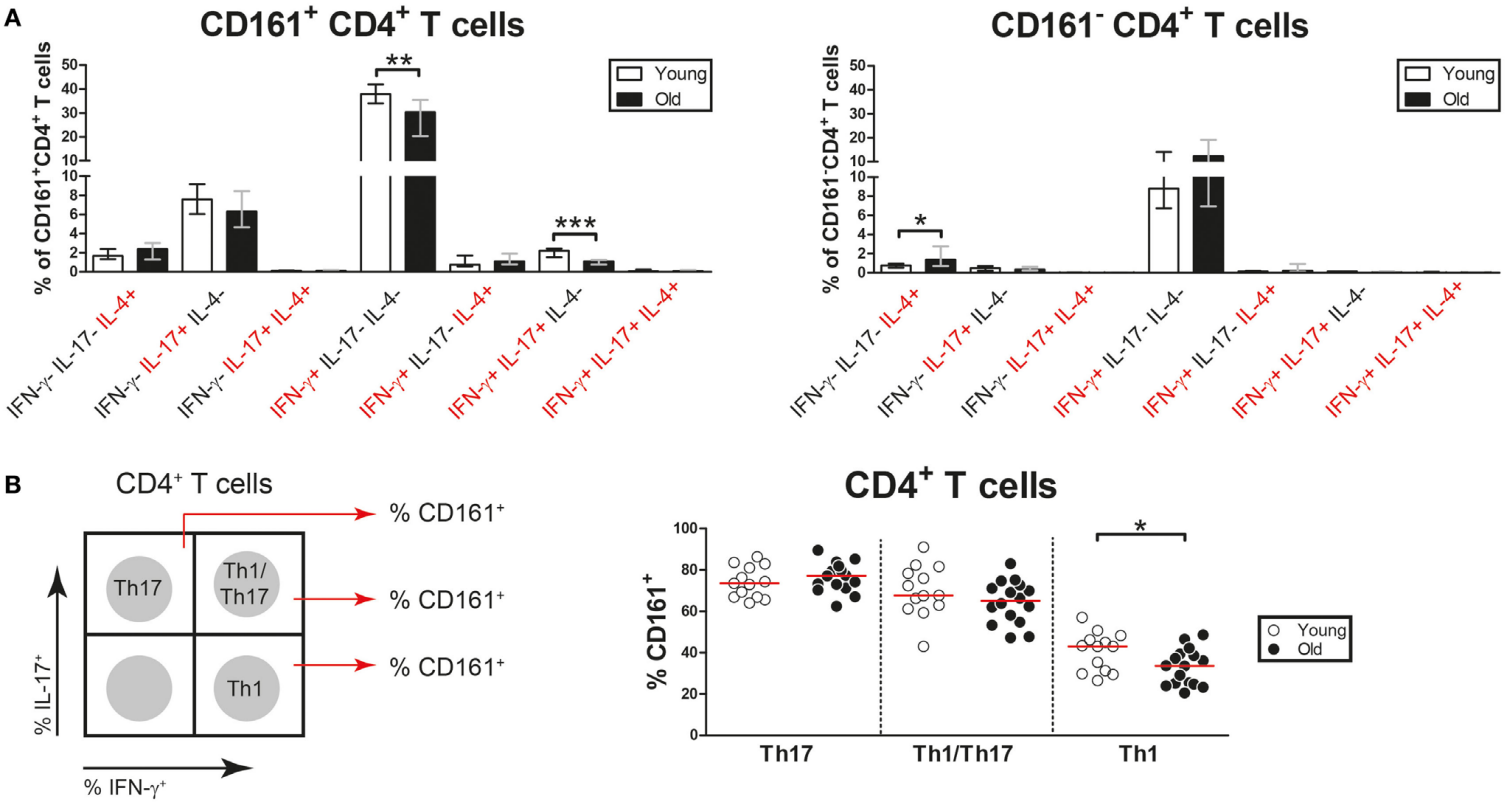
Co-expression of pro-inflammatory cytokines by CD4^+^ T cells. Intracellular staining for pro-inflammatory cytokines was determined on blood samples that were stimulated with PMA and calcium ionophore in the presence of brefeldin A. **(A)** Analysis of co-expression of IFN-γ, interleukin (IL)-17, and IL-4 by CD161^+^ CD4^+^ T cells and CD161^−^ CD4^+^ T cells of 13 young [of which 8 were cytomegalovirus (CMV) seropositive] and 23 old (of which 15 were CMV seropositive) subjects. Percentages of cells co-expressing different combinations of these cytokines within the CD161^+^ and CD161^−^ CD4^+^ T cell pool are shown. **(B)** Analysis of CD161 expression by Th17 cells (IL-17^+^ IFN-γ^−^), Th1/Th17 cells (IL-17^+^ IFN-γ^+^), and Th1 cells (IL-17^−^ IFN-γ^+^) in 13 young (of which 8 were CMV seropositive) and 16 old (of which 11 were CMV seropositive) subjects. A schematic illustration of the gating strategy is shown in the left panel. Percentages of CD161^+^ cells among Th17, Th1/Th17, and Th1 cells are shown in the right panel. Bars and whiskers indicate median and interquartile range. Statistical significance by Mann–Whitney *U*-test is shown as *p < 0.05 and **p < 0.01.

CD4^+^ T cells may co-express pro-inflammatory cytokines such as IFN-γ, IL-17, and IL-4 (39). Therefore, we next investigated CD161^+^ and CD161^−^ CD4^+^ T cells for the co-expression of the Th1, Th17, and Th2 lineage cytokines, i.e., IFN-γ, IL-17, and IL-4, respectively. The CD161^+^ CD4^+^ T cell pool contained cells solely producing IFN-γ, IL-17, or IL-4, but also IFN-γ^+^ IL-17^+^, and IFN-γ^+^ IL-4^+^ cells (Figure 8A). Interestingly, the percentages of both IFN-γ^+^ and IFN-γ^+^ IL-17^+^ cells were decreased among CD161^+^ CD4^+^ T cells of old subjects when compared to those of young subjects. In essence, the CD161^−^ CD4^+^ T cell pool showed limited potential to produce pro-inflammatory cytokines, with only few cells producing solely IFN-γ or IL-4. The percentages of IL-4-producing cells were slightly higher in CD161^−^ CD4^+^ T cells of old subjects than those of young subjects. Thus, the percentages of Th1 and Th1/Th17 cells are decreased in the CD161^+^ CD4^+^ T cell compartment of aged subjects.

Prior reports indicate that Th17 cells show plasticity toward Th1/Th17 cells and ultimately Th1 cells. The latter cells have been termed nonclassic Th1 cells and are characterized by the expression of CD161 (39). To assess the impact of aging on frequencies of nonclassic Th1 cells, we next investigated CD161 expression on Th1, Th1/Th17, and Th17 cells (Figure 8B, left panel). Percentages of CD161-expressing cells were equally high among Th17 and Th1/Th17 cells of young and old subjects (Figure 8B, right panel). Percentages of CD161-expressing cells were clearly lower among Th1 cells than among Th17 and Th1/Th17 cells. Moreover, fewer Th1 cells expressed CD161 in old subjects than in young subjects. This finding suggests that aging is associated with decreased frequencies of nonclassic Th1 cells.

## Discussion

We here provide the first comprehensive analysis of the impact of aging on CD161-expressing T cells in humans. We show that the numbers of CD161^high^ CD8^+^ T cells decline with age, whereas the numbers of CD161^int^ CD8^+^ T cells and CD161^+^ CD4^+^ T cells remain stable. In respect to the expression of innate-like T cell markers, differentiation markers, and NK receptors, the phenotype of CD161-expressing T cell subsets appeared rather stable with age. Overall, the expression of pro-inflammatory cytokines and cytotoxic effector molecules was comparable in CD161-expressing T cells of young and old subjects. However, the ability to produce IFN-γ and IL-17 upon stimulation was diminished among CD161^+^ CD4^+^ T cells of old individuals. Thus, aging is associated with both numerical and functional changes of CD161-expressing T cells, whereas we observed no substantial phenotypic alterations of these cells.

Aging was associated with stable frequencies of circulating CD161^+^ CD4^+^ T cells and a diminished production of pro-inflammatory cytokines by these cells. So far, the impact of age on the numbers of CD161^+^ CD4^+^ T cells remained unclear. The absolute numbers of CD161^+^ CD4^+^ T cells were not affected by aging in our multivariate linear regression analysis. We confirmed that CD161^+^ CD4^+^ T cells primarily show a CM or an EM phenotype, both in young and in old subjects. In accordance with their pro-inflammatory function, CD161^+^ CD4^+^ T cells produced more IFN-γ, IL-17, and TNF-α than CD161^−^ CD4^+^ T cells. Previously, we have shown that frequencies of Th17 cells and Th1 cells are decreased within the memory CD4^+^ T cell compartment of elderly subjects (8). In the current study, we add that the proportions of IL-17^+^ and IFN-γ^+^ cells are decreased among CD161^+^ CD4^+^ T cells of old subjects. Thus, the pro-inflammatory function of CD161^+^ CD4^+^ T cells declines with age.

Frequencies of nonclassic Th1 cells were found to be decreased in old subjects. Previous studies have shown that most Th17 cells are contained within the CD161^+^ CD4^+^ T cell compartment (18). It has been suggested that these Th17 cells show plasticity toward Th1/Th17 cells and eventually Th1 cells under pro-inflammatory conditions (30, 31). The latter Th1 cells have been termed nonclassic Th1 cells, while maintaining their expression of CD161 (39). In the current study, we show that the proportions of Th1/Th17 and Th1 cells are decreased among CD161^+^ CD4^+^ T cells of old subjects when compared to young subjects. Moreover, we show that fewer Th1 cells expressed CD161 in old subjects. Together, these findings suggest that aging affects the plasticity of Th17 cells toward nonclassic Th1 cells. It would be interesting to further study the mechanisms explaining this decreased plasticity in the elderly.

The numbers of circulating CD161^high^ CD8^+^ T cells decreased with age, whereas their ability to produce pro-inflammatory cytokines remained intact. As previously shown by others (32, 33), we observed that most CD161^high^ CD8^+^ T cells are MAIT cells, as evidenced by the expression of the TCR-Vα7.2 receptor. Proportions of MAIT cells were uniformly high among CD161^high^ CD8^+^ T cells of young and old subjects. In accordance with prior reports (34–36), we observed that the numbers of CD161^high^ TCR-Vα7.2^+^ CD8^+^ T cells decline with age. We confirmed that CD161^high^ CD8^+^ T cells are mostly contained within the EM compartment (19). Prior reports indicate that CD161^high^ CD8^+^ T cells are strong producers of pro-inflammatory cytokines (19). In the current study, we show that this function of CD161^high^ CD8^+^ T cells is not affected by aging. CD161^high^ CD8^+^ T cells of young and old subjects showed similar ability to produce IFN-γ, IL-17, and TNF-α. This finding seems in agreement with a prior study showing that the percentages of IFN-γ^+^ and IL-17^+^ cells are comparable in the CD8^+^ MAIT cell compartment of young and old individuals (34). Thus, only the frequency, but not the pro-inflammatory function, of CD161^high^ CD8^+^ T cells declines with age.

Neither the numbers of CD161^int^ CD8^+^ T cells nor their cytokine-producing potential was affected by age. The absolute numbers of CD161^int^ CD8^+^ T cells were comparable in young and old subjects. Nevertheless, the percentages of CD161^int^ CD8^+^ T cells showed an increase with age due to loss of CD161^high^ and CD161^−^ CD8^+^ T cells from the CD8^+^ T cell compartment. CD161^int^ CD8^+^ T cells primarily resided within the EM and TD compartments, as previously shown by others (20). In this respect, we observed no difference between CD161^int^ CD8^+^ T cells of young and old subjects. We confirmed that most CD161^int^ CD8^+^ T cells are able to produce IFN-γ and TNF-α (19, 20). In addition, CD161^int^ CD8^+^ T cells of old subjects more frequently produced IFN-γ than those of young subjects, whereas no difference was observed for TNF-α. Thus, both the frequencies and cytokine-producing potential of CD161^int^ CD8^+^ T cells are preserved on to high age.

The expression of NK receptors was comparable in CD161-expressing T cells of young and old subjects. In addition to signaling *via* the TCR and conventional co-stimulation molecules, T cell activation may be influenced by NK receptors. In particular, late-stage T cells of aged subjects may express activating and inhibitory NK receptors (6, 7). We here examined CD161-expressing T cells for the presence of three well-defined activating NK receptors (i.e., 2B4, DNAM-1, and NKG2D), as well as one inhibitory NK receptor (i.e., KLRG1). CD161^high^ and CD161^int^ CD8^+^ T cells showed prominent expression of all four NK receptors, without any difference between young and old subjects. By contrast, CD161^+^ CD4^+^ T cells primarily expressed DNAM-1 and KLRG1. DNAM-1 expression was similar in CD161^+^ CD4^+^ T cells of young and old subjects, but the percentage of KLRG1^+^ cells was decreased among CD161^+^ CD4^+^ T cells of old subjects. Although our analysis was restricted to only four NK receptors, a decreased expression of the latter inhibitory NK receptor could indicate that CD161^+^ CD4^+^ T cells of old subjects might be more prone to activation.

The expression of cytotoxic effector molecules by CD161-expressing T cells was not affected by age. CD161^+^ CD4^+^ T cells showed little expression of perforin and granzyme B, irrespective of age. Approximately half of the CD161^int^ CD8^+^ T cells expressed perforin and granzyme B in young and old subjects. This finding underscores the prominent cytotoxic potential of these cells. Similar percentages of perforin expressing CD161^high^ CD8^+^ T cells were observed in young and old individuals. In accordance with prior studies, few CD161^high^ CD8^+^ T cells expressed granzyme B (19, 40), both in young and in old subjects. It has been demonstrated that CD161^high^ CD8^+^ T cells primarily express granzymes A and K (40). Although the latter cytotoxic effector molecules were not analyzed in the current study, the stable expression of perforin by CD161^high^ CD8^+^ T cells suggests that the cytotoxic potential of these cells remains intact with age.

Limited data suggest that CD161-mediated signaling promotes the secretion of pro-inflammatory cytokines by T cells. Lectin-like transcript 1 (LLT1) has been identified as the ligand for CD161 (41, 42). LLT1 is expressed by antigen-presenting cells, including B cells and macrophages (43, 44). It has been shown that the engagement of CD161 by LLT1 enhances the production of pro-inflammatory cytokines by T cells. For instance, the ligation of CD161 when given in addition to TCR stimulation increased the production of IFN-γ and TNF-α by MAIT cells (37). Similar experiments with CD161-expressing T cell clones also indicate that CD161 ligation in the presence of TCR stimulation promotes IFN-γ production by T cells (41). Thus, current evidence suggests that CD161 may act as a co-stimulatory receptor on T cells. An early study has also suggested that CD161 may be directly involved in transendothelial migration of T cells (45). However, this observation has not yet been confirmed by others. It would be interesting to study the effect of aging on the function of CD161 itself.

Given the broad antimicrobial functions of CD161-expressing T cells (20–24), it is likely that the aging-associated changes of CD161^+^ CD4^+^ T cells and CD161^high^ CD8^+^ T cells may compromise immunity in the elderly. The CD161^+^ CD4^+^ T cell compartment contains antiviral Th1-like cells (22), as well as Th17 cells promoting immunity against bacteria and yeasts (46). In patients undergoing treatment for hematological malignancies, CD161^+^ CD4^+^ T cells have recently been linked to preserved immunity against CMV and lower risks for neutropenic infections (22, 47). CD161^high^ CD8^+^ T cells, which primarily consist of MAIT cells, are critical for immunity against common bacteria and viruses, such as *Escherichia coli* and influenza, respectively (23, 48). Numerical decreases of MAIT cells might put elderly subjects at risk for infections with these microbes. Indeed, sepsis due to Gram-negative bacteria and pneumonia occurs more frequently and with a higher severity in the elderly (49). The CD161^int^ CD8^+^ T cell compartment has been identified as a polyclonal CD8^+^ T cell population that contributes to antiviral immunity (20). The stable frequencies of CD161^int^ CD8^+^ T cells, and the ability to produce cytotoxic effector molecules and pro-inflammatory cytokines, likely contribute to immunity throughout adult life. Thus, not all CD161-expressing T cell subsets are compromised with age. It remains to be elucidated if the aging-related effects on CD161^+^ CD4^+^ T cells and CD161^high^ CD8^+^ T cells might be advantageous in the context of autoimmune diseases (25, 26, 50).

We precluded that CMV confounded our findings regarding the impact of aging on CD161-expressing T cells. Indeed, CMV markedly influences the T cell compartment and might potentially compromise immunity to other pathogens in the elderly (9–11, 17). Therefore, we delineated the effects of aging and CMV on the absolute numbers of CD161-expressing cells by performing multivariate linear regression analyses. We confirmed that the aging-associated decline of CD161^high^ CD8^+^ T cell numbers occurred independently from CMV serostatus. These analyses also demonstrated that CMV seropositivity by itself is associated with higher numbers of CD161^int^ CD8^+^ T cells. Interestingly, a recent report indicates that CD161^int^ CD8^+^ T cells are highly functional memory cells that contribute to antiviral immunity (20). Ample evidence also suggests that CMV-specific memory cells upregulate NK receptors and have a strong potential to produce cytotoxic effector molecules and pro-inflammatory cytokines (12–14). Importantly, CMV-seropositivity rates were comparable between young and old subjects in our extensive phenotypical and functional analyses of CD161-expressing T cells. Thus, CMV unlikely influenced the findings in the current study. Indeed, it would be interesting to further perform a comprehensive analysis of CMV and CD161-expressing T cells.

In conclusion, we here show that aging affects the frequencies and function of CD161-expressing T cells. Aging-associated changes of CD161-expressing T cells might potentially contribute to a decreased antimicrobial immunity in the elderly. Future studies should evaluate the mechanisms and implications of aging-associated changes of CD161-expressing T cells in humans.

## Ethics Statement

Written informed consent was obtained from all study participants. The study was approved by the Medical Ethical Committee of the UMCG. All procedures were in accordance with the Declaration of Helsinki.

## Author Contributions

KG, B-JK, WA, EB, and AB conceived the study and designed the experiments. KG and EB recruited the study participants. KG and GH performed the experiments and acquired data. All authors were involved in data analysis and interpretation. KG and AB wrote the manuscript, and all authors revised it critically for important intellectual content. All authors approved the final version of the manuscript.

## Conflict of Interest Statement

The authors declare that the research was conducted in the absence of any commercial or financial relationships that could be construed as a potential conflict of interest.

## Abbreviations

CM, central memory; CMV, cytomegalovirus; DN, double negative; DNAM-1, DNAX accessory molecule-1; EM, effector memory; IL, interleukin; LLT1, lectin-like transcript 1; iNKT cell, invariant natural killer T cell; MAIT cell, mucosal-associated invariant T cell; NK, natural killer; TCR, T cell receptor; TD, terminally differentiated; Th1, T helper 1; Th17, T helper 17; TNF-α, tumor necrosis factor-α.
