# Supplementary material for: Impact of Aging on the Frequency, Phenotype, and Function of CD161-Expressing T Cells
Source: Front Immunol. 2018 Apr 19;9:752. doi: 10.3389/fimmu.2018.00752 (PMC5917671; doi:10.3389/fimmu.2018.00752)

Supplementary Figure 1. Effect of age on proportions of CD161 expressing T cells. (A) Percentages of $CD161^+$ $CD4^+$ T cells and $CD161^-$ $CD4^+$ T cells within the circulating CD4+ T cell compartment of 96 healthy subjects (age range 20-84). (B) Percentages of $CD161^{high}$ $CD8^+$ T cells, $CD161^{int}$ $CD8^+$ T cells and $CD161^-$ $CD8^+$ T cells within the circulating CD8+ T cell compartment of the same donors as mentioned at (A). Statistical significance by Spearman's rank test is shown.

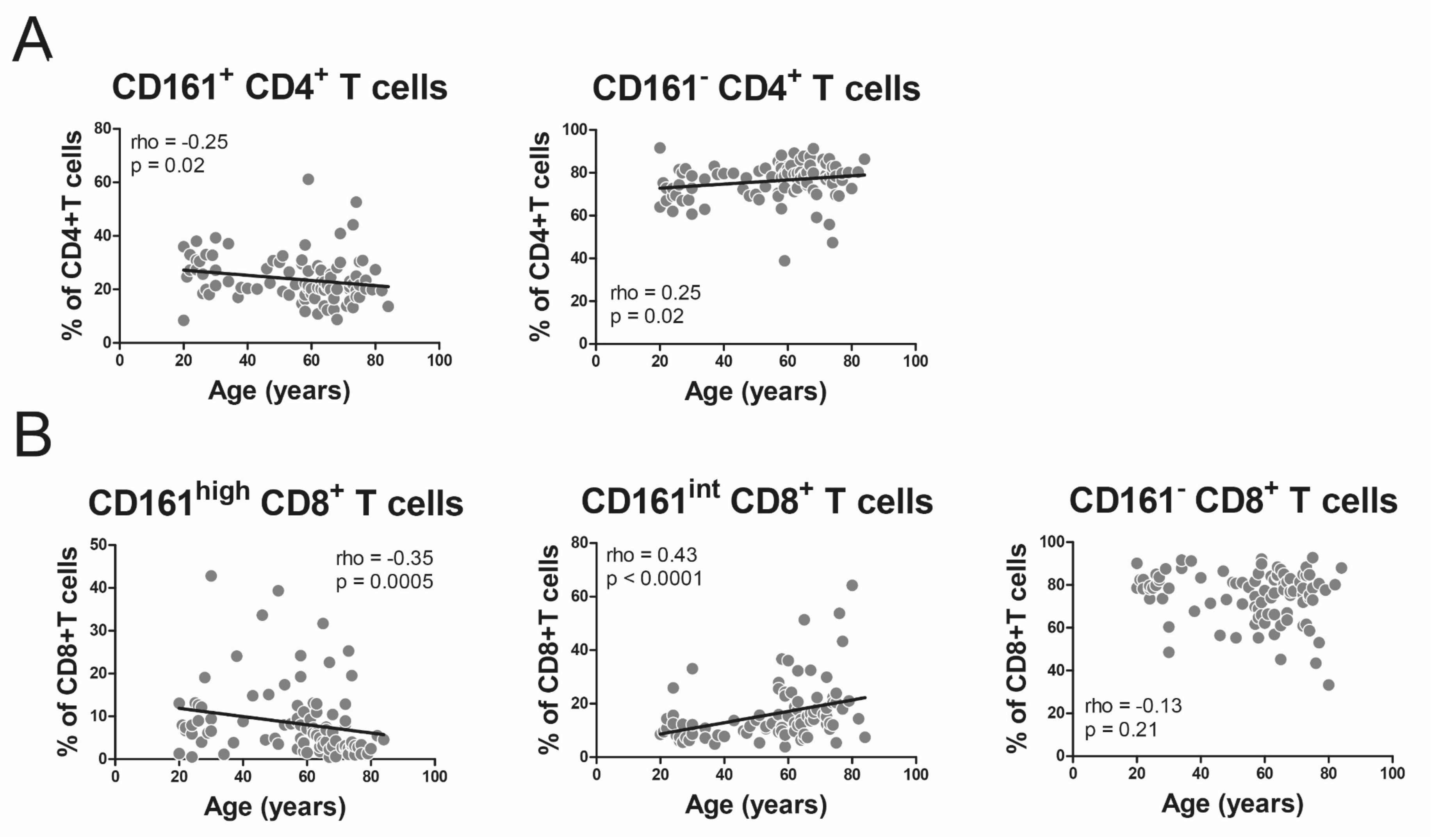

Supplement: Supplementary file 1 [file image_1.PDF]
