# Supplementary material for: Impact of Aging on the Frequency, Phenotype, and Function of CD161-Expressing T Cells
Source: Front Immunol. 2018 Apr 19;9:752. doi: 10.3389/fimmu.2018.00752 (PMC5917671; doi:10.3389/fimmu.2018.00752)

Supplementary Figure 2. Innate-like T cell markers on CD161 expressing T cells. (A) Percentages of TCRγδ$^+$ cells within the CD161-defined CD4$^+$ and CD8$^+$ T cell subsets of 7 young (of which 4 CMV seropositive) and 16 old (of which 8 CMV seropositive) subjects. (B) Percentages of TCR-Vα24Jα18$^+$ TCR-Vβ11$^+$ cells within the CD161-defined CD4$^+$ and CD8$^+$ T cell subsets of 9 young (of which 4 CMV seropositive) and 9 old (of which 4 CMV seropositive) subjects. (C) Percentages of TCR-Vα7.2$^+$ cells within the CD161-defined CD4$^+$ and CD8$^+$ T cell subsets of 10 young (of which 5 CMV seropositive) and 10 old (of which 5 CMV seropositive) subjects. White dots represent CMV seronegative subjects. Red dots represent CMV seropositive subjects.

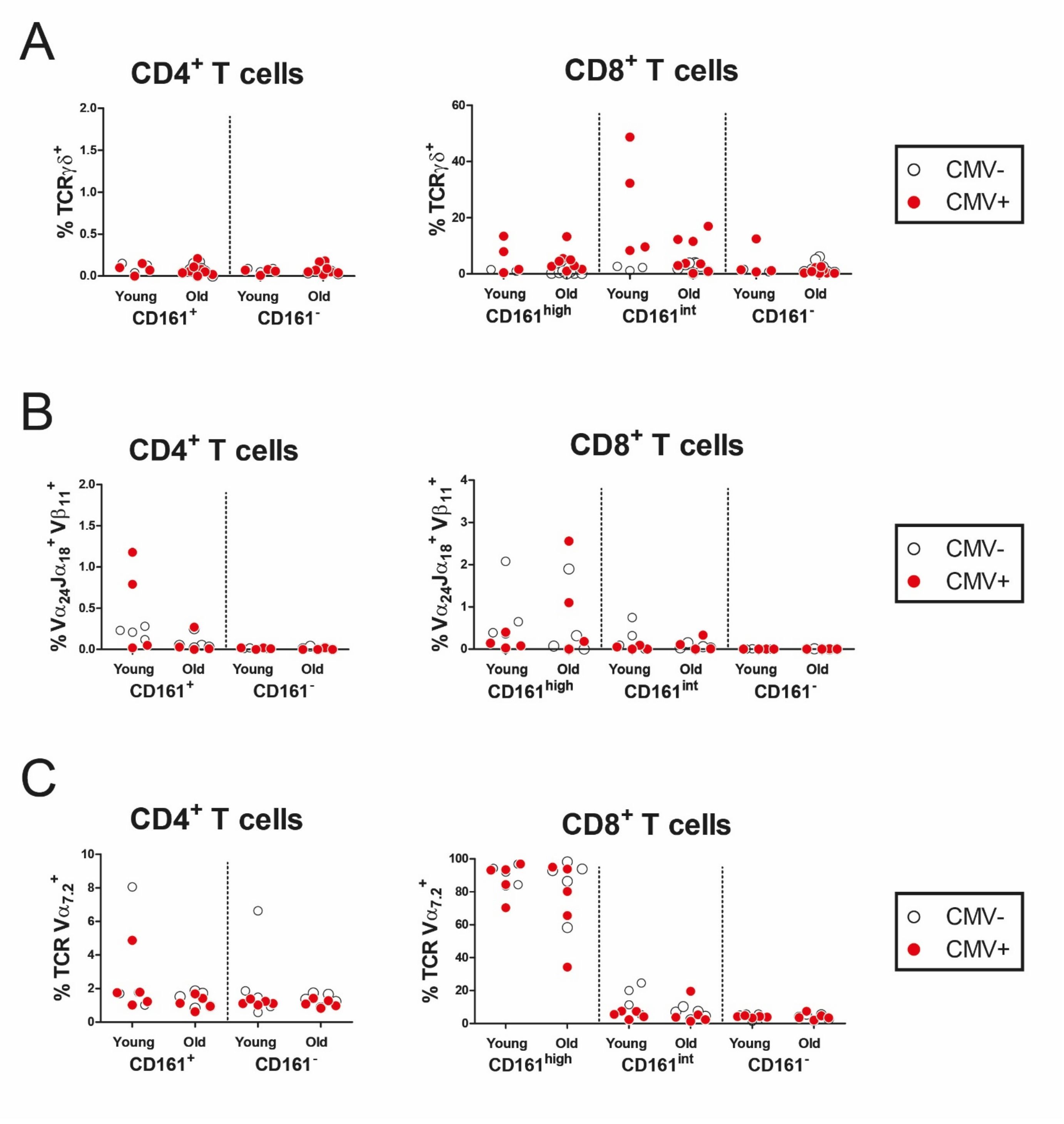

Supplement: Supplementary file 2 [file image_2.PDF]
