# Supplementary material for: Impact of Aging on the Frequency, Phenotype, and Function of CD161-Expressing T Cells
Source: Front Immunol. 2018 Apr 19;9:752. doi: 10.3389/fimmu.2018.00752 (PMC5917671; doi:10.3389/fimmu.2018.00752)

Supplementary Figure 3. Proportions of innate-like T cells in the CD3^{+} T cell compartment (A) Percentages of total TCRγδ^{+} cells (i.e. gamma-delta T cells), (B, left panel) CD161^{+} CD8^{+} gamma-delta T cells, (B right panel) CD161^{-} CD8^{+} gamma-delta T cells, (C, left panel) CD161^{+} double negative (DN) gamma-delta T cells and (C, right panel) CD161^{-} DN gamma-delta T cells within the CD3^{+} T cell compartment of 7 young (of which 4 CMV seropositive) and 16 old (of which 8 CMV seropositive) subjects. (D) Percentages of TCR-Vα24Jα18^{+}TCR-Vβ11^{+} cells (i.e. invariant natural killer T cells) within the CD3^{+} T cell compartment of 9 young (of which 4 CMV seropositive) and 9 old (of which 4 CMV seropositive) subjects. (E) Percentages of total CD161^{high} TCR-Vα7.2^{+} mucosal-associated invariant T (MAIT) cells, (F, left panel) CD8^{+} MAIT cells and (F, right panel) DN MAIT cells within the CD3^{+} T cell compartment of 10 young (of which 5 CMV seropositive) and 10 old (of which 5 CMV seropositive) subjects. White dots represent CMV seronegative subjects. Red dots represent CMV seropositive subjects.

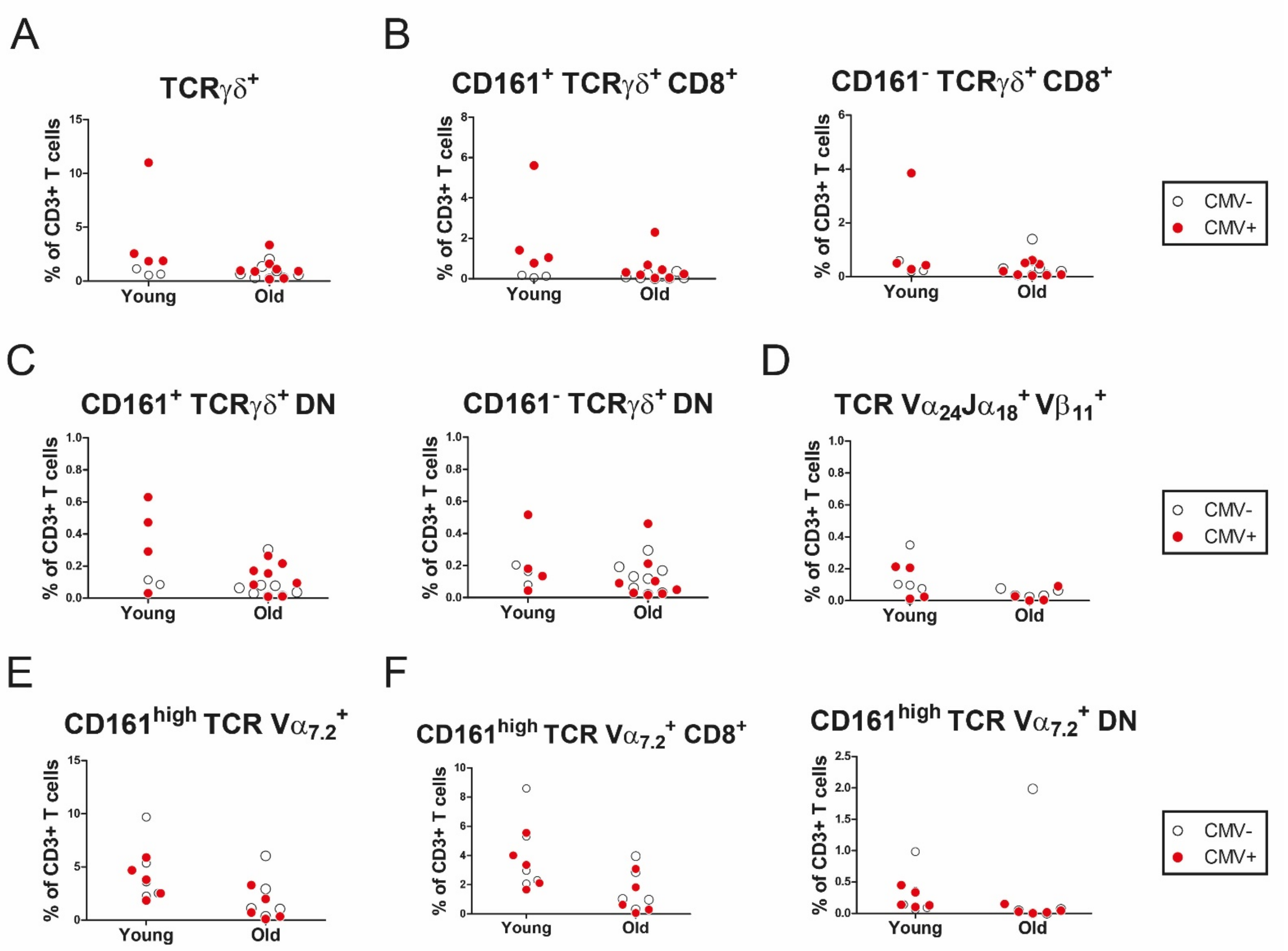

Supplement: Supplementary file 3 [file image_3.PDF]
