# Supplementary material for: Impact of Aging on the Frequency, Phenotype, and Function of CD161-Expressing T Cells
Source: Front Immunol. 2018 Apr 19;9:752. doi: 10.3389/fimmu.2018.00752 (PMC5917671; doi:10.3389/fimmu.2018.00752)

Supplementary Figure 4. Differentiation markers on CD161 expressing T cells. (A) Percentages of differentiation subsets within the CD161-defined $CD4^+$ T cell subsets of 20 young (of which 10 CMV seropositive) and 44 old (of which 22 CMV seropositive) subjects. (B) Percentages of differentiation subsets within the CD161-defined $CD8^+$ T cell subsets of the same donors as mentioned at (A). White dots represent CMV seronegative subjects. Red dots represent CMV seropositive subjects.

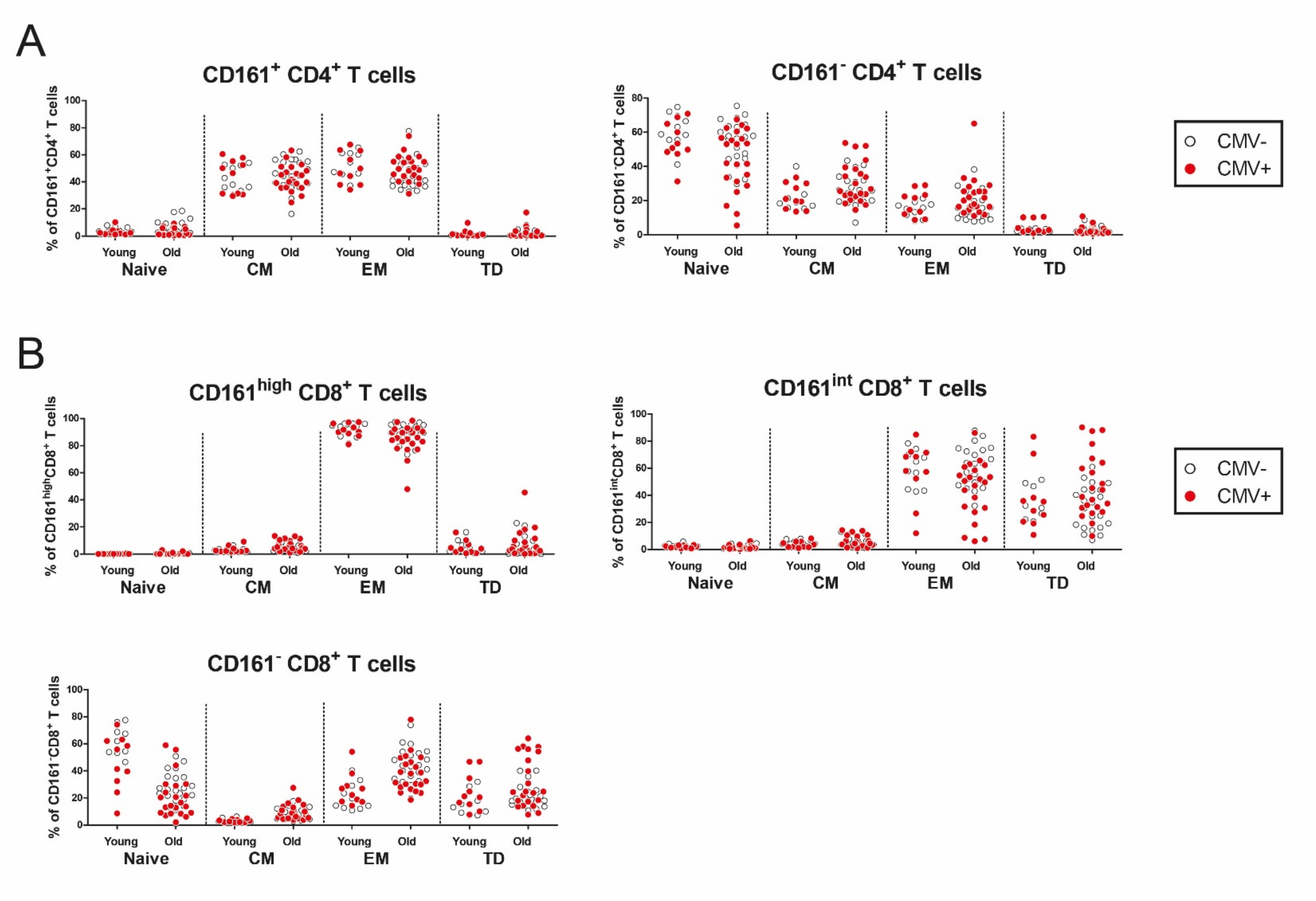

Supplement: Supplementary file 4 [file image_4.PDF]
