# Supplementary material for: Impact of Aging on the Frequency, Phenotype, and Function of CD161-Expressing T Cells
Source: Front Immunol. 2018 Apr 19;9:752. doi: 10.3389/fimmu.2018.00752 (PMC5917671; doi:10.3389/fimmu.2018.00752)

Supplementary Figure 5. Natural killer markers on CD161 expressing T cells. (A) Percentages of 2B4$^+$ cells within the CD161-defined CD4$^+$ and CD8$^+$ T cell subsets of 8 young (of which 4 CMV seropositive) and 15 old (of which 7 CMV seropositive) subjects. (B) Percentages of DNAM-1$^+$ cells within the CD161-defined CD4$^+$ and CD8$^+$ T cell subsets the same subjects as mentioned at (A). (C) Percentages of NKG2D$^+$ cells within the CD161-defined CD4$^+$ and CD8$^+$ T cell subsets of 11 young (of which 6 CMV seropositive) and 14 old (of which 8 CMV seropositive) subjects. (D) Percentages of KLRG1$^+$ cells within the CD161-defined CD4$^+$ and CD8$^+$ T cell subsets of the same subjects as mentioned at (C). White dots represent CMV seronegative subjects. Red dots represent CMV seropositive subjects.

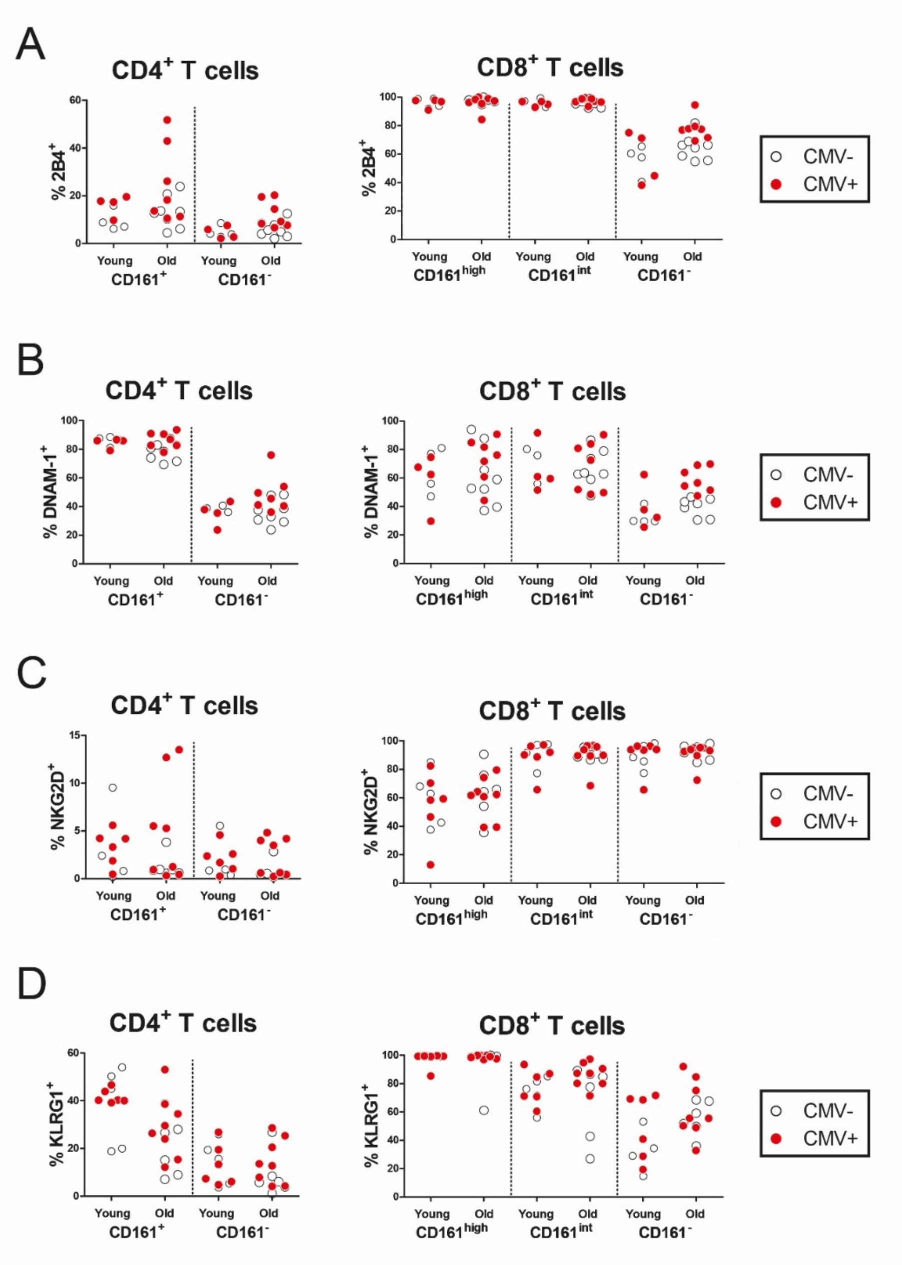

Supplement: Supplementary file 5 [file image_5.PDF]
