# Supplementary material for: Impact of Aging on the Frequency, Phenotype, and Function of CD161-Expressing T Cells
Source: Front Immunol. 2018 Apr 19;9:752. doi: 10.3389/fimmu.2018.00752 (PMC5917671; doi:10.3389/fimmu.2018.00752)

Supplementary Figure 6. Cytotoxic effector molecules in CD161 expressing T cells. Intracellular staining for perforin and granzyme B was performed on non-stimulated blood samples. (A) Percentages of perforin$^+$ cells within the CD161-defined CD4$^+$ and CD8$^+$ T cell subsets of 11 young (of which 7 CMV seropositive) and 12 old (of which 8 CMV seropositive) subjects. (B) Percentages of granzyme B$^+$ cells within the CD161-defined CD4$^+$ and CD8$^+$ T cell subsets of the same subjects as mentioned at (A). White dots represent CMV seronegative subjects. Red dots represent CMV seropositive subjects.

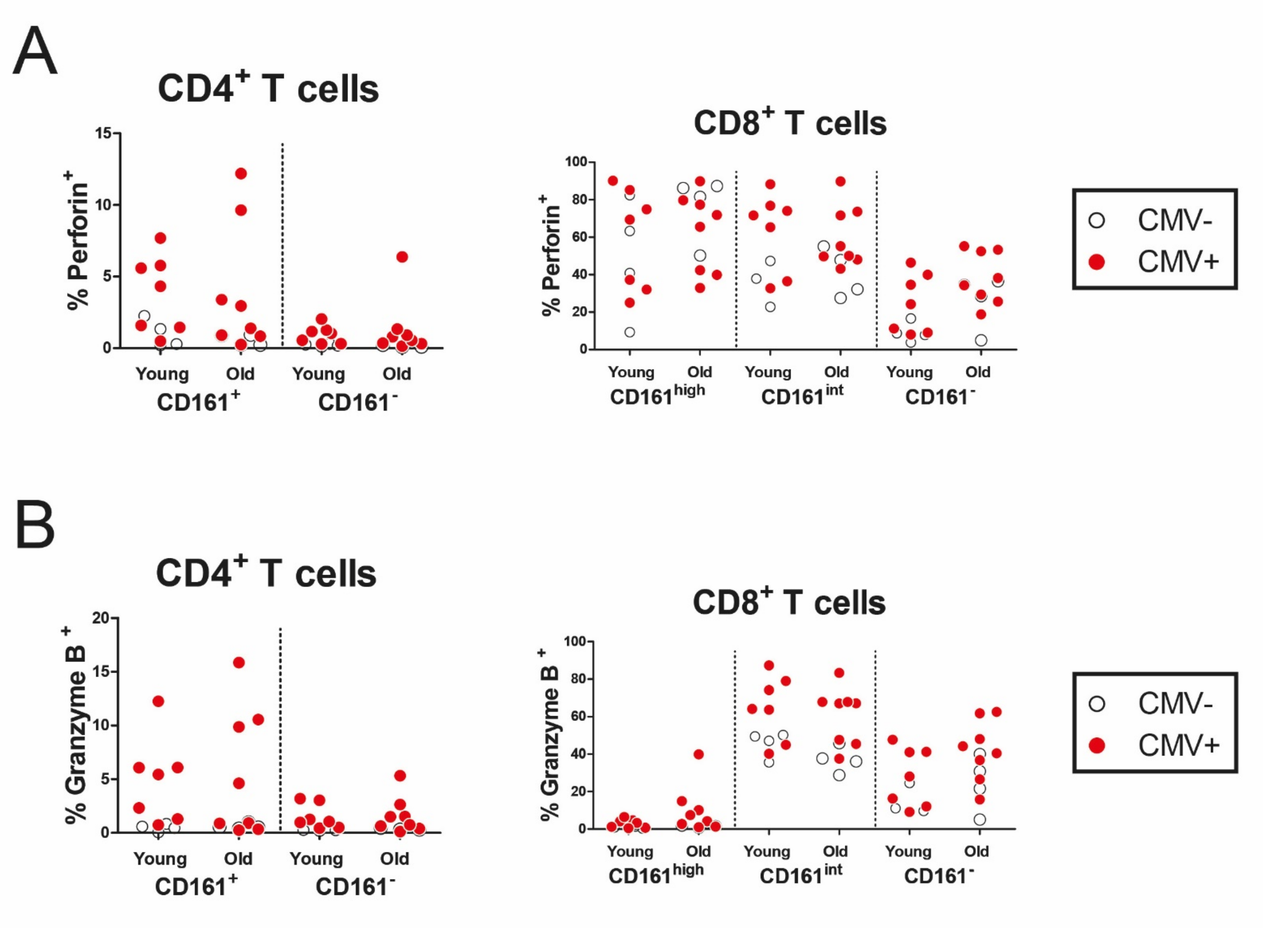

Supplement: Supplementary file 6 [file image_6.PDF]
