# Supplementary material for: Impact of Aging on the Frequency, Phenotype, and Function of CD161-Expressing T Cells
Source: Front Immunol. 2018 Apr 19;9:752. doi: 10.3389/fimmu.2018.00752 (PMC5917671; doi:10.3389/fimmu.2018.00752)

frontiers

Supplementary Figure 7. Cytokine expression by CD161 expressing T cells. Intracellular staining for pro-inflammatory cytokines was determined on blood samples that were stimulated with PMA and calcium ionophore in the presence of brefeldin A. (A) Percentages of IFN-γ^+ cells within the CD161-defined $CD4^+$ T cell subsets of 13 young (of which 8 CMV seropositive) and 23 old (of which 15 CMV seropositive) subjects. (B) Percentages of IFN-γ^+ cells within the CD161-defined $CD8^+$ T cell subsets of 13 young (of which 8 CMV seropositive) and 18 old (of which 12 CMV seropositive) subjects. (C) Percentages of IL-17^+ cells within the CD161-defined $CD4^+$ T cell subsets of the same subjects as shown in (A). (D) Percentages of IL-17^+ cells within the CD161-defined $CD8^+$ T cell subsets of the same subjects as shown in (B). (E) Percentages of IL-4^+ cells within the CD161-defined $CD4^+$ T cell subsets of the same subjects as shown in (A). (F) Percentages of IL-4^+ cells within the CD161-defined $CD8^+$ T cell subsets of the same subjects as shown in (B). (G) Percentages of TNF-α^+ cells within the CD161-defined $CD4^+$ T cell subsets and (H) CD161-defined $CD8^+$ T cell subsets of 12 young (of which 7 CMV seropositive) and 12 old (of which 7 CMV seropositive) subjects. White dots represent CMV seronegative subjects. Red dots represent CMV seropositive subjects.

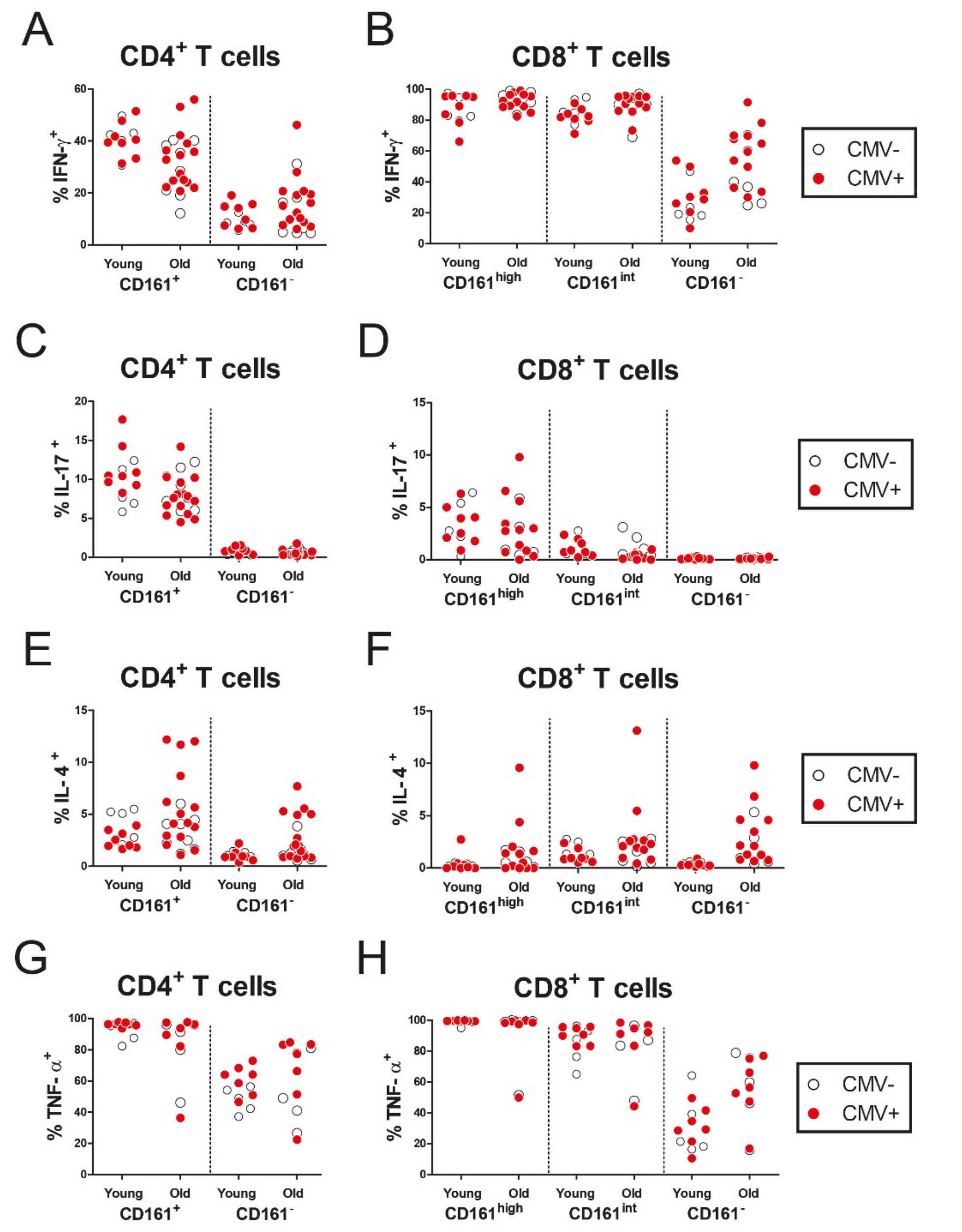

Supplement: Supplementary file 7 [file image_7.PDF]
