# Supplementary material for: Impact of Aging on the Frequency, Phenotype, and Function of CD161-Expressing T Cells
Source: Front Immunol. 2018 Apr 19;9:752. doi: 10.3389/fimmu.2018.00752 (PMC5917671; doi:10.3389/fimmu.2018.00752)
Supplement: Supplementary file 8 [file table_1.PDF]

Supplementary Table 1. Overview of monoclonal antibody panels for flow cytometry.

Panel 1	Panel 2	Panel 3	Panel 4
CD3-eFluor605<br>CD4-eFluor450<br>CD8-PerCP<br>CD161-PE<br>CD45RO-FITC<br>CCR7-PE-Cy7	CD3-eFluor605<br>CD4-eFluor450<br>CD8-APC-H7<br>CD161-APC<br>TCRγδ-PE	CD3-eFluor605<br>CD4-eFluor450<br>CD8-APC-H7<br>CD161-APC<br>TCR-Vα24-Jα18-FITC<br>TCR-Vβ11-PE	CD3-eFluor605<br>CD4-eFluor450<br>CD8-APC-H7<br>CD161-PE<br>TCR-Vα7.2-FITC

Panel 5	Panel 6	Panel 7
CD3-eFluor605<br>CD4-eFluor450<br>CD8-APC-H7<br>CD161-APC<br>DNAM1-FITC<br>2B4-PE	CD3-eFluor605<br>CD4-eFluor450<br>CD8-APC-H7<br>CD161-APC<br>NKG2D-PE-Cy7	CD3-eFluor605<br>CD4-PerCP<br>CD8-APC-H7<br>CD161-APC<br>KLRG1-FITC

Panel 8	Panel 9
CD3-eFluor605<br>CD4-eFluor450<br>CD8-APC-H7<br>CD161-APC<br>TNF-α-PerCP-Cy5.5<br>Granzyme B-PE<br>Perforin-FITC	CD3-eFluor605<br>CD4-eFluor450<br>CD8-APC-H7<br>CD161-APC<br>IFN-γ-PerCP-Cy5.5<br>IL-4-PE<br>IL-17-AF488
