# Supplementary material for: Impact of Aging on the Frequency, Phenotype, and Function of CD161-Expressing T Cells
Source: Front Immunol. 2018 Apr 19;9:752. doi: 10.3389/fimmu.2018.00752 (PMC5917671; doi:10.3389/fimmu.2018.00752)
Supplement: Supplementary file 9 [file table_2.PDF]

Supplementary Table 2. CMV serostatus of healthy young and old subjects in the experiments reporting phenotype and function of CD161 expressing T cells. No. = number. CMV+ = CMV seropositive.

**Figure**	**Analysis**	**Young donors**		**Old donors**	
		Total no.	*No. CMV+ (%)*	*Total no.*	*No. CMV+ (%)*
2C	TCRγδ	7	4 (57%)	16	8 (50%)
2D	TCR Vα24Jα18 TCR Vβ11	9	4 (44%)	9	4 (44%)
2F	TCR Vα7.2	10	5 (50%)	10	5 (50%)
3A, B and C	TCRγδ	7	4 (57%)	16	8 (50%)
2D	TCR Vα24Jα18 TCR Vβ11	9	4 (44%)	9	4 (44%)
2E and F	TCR Vα7.2	10	5 (50%)	10	5 (50%)
4B and C	CD45RO and CCR7	20	10 (50%)	44	22 (50%)
5B and D	2B4 and DNAM-1	8	4 (50%)	15	7 (47%)
5F and H	NKG2D and KLRG1	11	6 (55%)	14	8 (57%)
6B and D	Perforin and Granzyme B	11	7 (64%)	12	8 (67%)
7B, E and H	IFN-γ, IL-17 and IL-4	13	8 (62%)	23	15 (65%)
7C, F and I	IFN-γ, IL-17 and IL-4	13	8 (62%)	18	12 (67%)
7H	TNF-α	12	7 (58%)	12	7 (58%)
8A	IFN-γ, IL-17 and IL-4	13	8 (62%)	23	15 (65%)
8B	IFN-γ, IL-17 and IL-4	13	8 (62%)	16	11 (69%)
